# Correction: Influence of women’s legal status on pregnancy outcomes and quality of care: Findings from the Pregnancy of Migrants in Switzerland (PROMISES) program

**DOI:** 10.1371/journal.pgph.0005303

**Published:** 2025-10-09

**Authors:** Eugénie de Weck, Clara Noble, Jessica Sormani, Monique Lamuela Naulin, Cyril Jaksic, Sara Arsever, Begoña Martinez de Tejada, Nicole C. Schmidt, Anya Levy Guyer, Anne-Caroline Benski

The first two authors, Eugénie de Weck and Clara Noble, should be noted as shared first co-authors on this work.
