# Supplementary material for: Influence of women’s legal status on pregnancy outcomes and quality of care: Findings from the Pregnancy of Migrants in Switzerland (PROMISES) program
Source: PLOS Glob Public Health. 2025 Apr 21;5(4):e0004217. doi: 10.1371/journal.pgph.0004217 (PMC12011233; doi:10.1371/journal.pgph.0004217)
Supplement: S1 Table — (DOCX) [file pgph.0004217.s001.docx]

### Table 1: Independent variables

| **Variable** | **Options** | **Source data** |
| --- | --- | --- |
| *Migration status* | Migrant (M)  Swiss (S) | Electronic medical records and administrative files |
| *Legal status* | Documented (D)  Undocumented (U) | Electronic medical records and administrative files |
| *Precarity** | Precarious (P)  Non-precarious (NP) | All UMs and ASs were characterized as precarious.  For Swiss and documented migrants, precarity was calculated as follows:  Patients’ residential addresses were used to generate GPS coordinates (using the Nominatim search engine from the open-source software OpenStreetMap, available under the Open Database License) [32].  Using data provided by the Territory Information System in Geneva (SITG) (accessed on February 1, 2023) [33], the GPS coordinates were linked with one of the 475 sub-sectors in Geneva canton and assigned the median income for a single adult living in that sub-sector.  Precarity was then defined as below the defined cantonal minimum wage. |
