# Supplementary material for: Influence of women’s legal status on pregnancy outcomes and quality of care: Findings from the Pregnancy of Migrants in Switzerland (PROMISES) program
Source: PLOS Glob Public Health. 2025 Apr 21;5(4):e0004217. doi: 10.1371/journal.pgph.0004217 (PMC12011233; doi:10.1371/journal.pgph.0004217)
Supplement: S2 Table — (DOCX) [file pgph.0004217.s002.docx]

**Table 2: Demographic and dependent variables**

| **Category** | **Variable** | **Options (continuous or discrete)** | **Reason for inclusion** |
| --- | --- | --- | --- |
| **Socio-demographic variables** | *Age of patient* | Continuous | Known association with health outcomes & data available from records |
|  | *Parity* | Continuous | Known association with health outcomes & data available from records |
|  | *Gravidity* | Continuous | Known association with health outcomes & data available from records |
|  | *Nationality (Swiss or by continent)* | Discrete | Data available from records |
|  | *Tariff attribute* | Discrete | Data available from records |
|  | *French language acquisition* | Discrete | Data available from records |
|  | *Marital status* | Discrete | Data available from records |
|  | *Religion* | Discrete | Data available from records |
|  | *Profession* | Discrete | Data available from records |
|  | *Active smoker* | Discrete | Known association with health outcomes & data available from records |
| **Obstetrical variables** | *Term* | Discrete | Known association with health outcomes & data available from records |
|  | *Delivery type* | Discrete | Known association with health outcomes & data available from records |
|  | *Induced labor* | Discrete | Known association with health outcomes & data available from records |
|  | *Past C-section* | Discrete | Known association with health outcomes & data available from records |
|  | *Single or multiple pregnancy* | Discrete | Known association with health outcomes & data available from records |
|  | *Anesthesia* | Discrete | Data available from records |
|  | *Postpartum hemorrhage* | Discrete | Known association with health outcomes & data available from records |
|  | *Perineal tear* | Discrete | Known association with health outcomes & data available from records |
|  | *Episiotomy* | Discrete | Known association with health outcomes & data available from records |
|  | *Threat of preterm delivery* | Discrete | Known association with health outcomes & data available from records |
|  | *Gestational diabetes or diabetes* | Discrete | Known association with health outcomes & data available from records |
|  | *Gestational hypertension* | Discrete | Data available from records |
|  | *Eclampsia or preeclampsia* | Discrete | Known association with health outcomes & data available from records |
|  | *Female genital cutting* | Discrete | Known association with health outcomes & data available from records |
|  | *Newborn’s birth weight* | Continuous | Known association with health outcomes & data available from records |
|  | *Newborn’s hospitalization in the neonatal unit - main diagnosis* | Discrete | Known association with health outcomes & data available from records |
| **Quality variables** | *First contact: with hospital emergency room* | Discrete | Data available from records |
|  | *Private gynecological monitoring* | Discrete | Data available from records |
|  | *Folic acid supplementation* | Discrete | Known association with health outcomes & data available from records |
|  | *Breastfeeding* | Discrete | Known association with health outcomes & data available from records |
|  | *Admission motive* | Discrete | Data available from records |
|  | *Timely ultrasound* | Discrete | Data available from records |
|  | *Appropriate time for the first contact for the UMs* | Discrete | Data available from records |
