# Supplementary material for: Influence of women’s legal status on pregnancy outcomes and quality of care: Findings from the Pregnancy of Migrants in Switzerland (PROMISES) program
Source: PLOS Glob Public Health. 2025 Apr 21;5(4):e0004217. doi: 10.1371/journal.pgph.0004217 (PMC12011233; doi:10.1371/journal.pgph.0004217)
Supplement: S3 Table — (DOCX) [file pgph.0004217.s003.docx]

### Table 3: Sociodemographic variables, by group

| **Socio-demographic variable** | **Total**  **(n= 296)** | **Swiss non-precarious SNP**  **(n= 75, 25.3%)** | **Swiss precarious SP**  **(n=36, 12.2%)** | **Documented migrant non-precarious DMNP**  **(n=103, 34.8%)** | **Documented migrant precarious DMP**  **(n=69, 23.3%)** | **Undocumented migrants UM**  **(n=7, 2.4%)** | **Asylum seekers AS**  **(n=6, 2.0%)** |
| --- | --- | --- | --- | --- | --- | --- | --- |
| Age of patient |  |  |  |  |  |  |  |
|  | 31 (28 - 36) | 31 (28 - 35) | 31 (29 - 34.2) | 32 (29 - 36) | 32 (27 - 37) | 26 (25 - 33.5) | 29 (27.5 - 30.5) |
| Parity |  |  |  |  |  |  |  |
| 0 | 134 (45.3%) | 42 (53.3%) | 16 (44.4%) | 47 (45.6%) | 26 (37.7%) | 4 (57.1%) | 1 (16.7%) |
| 1 | 101 (34.1%) | 26 (34.7%) | 8 (22.2%) | 38 (36.9%) | 25 (36.2%) | 2 (28.6%) | 2 (33.3%) |
| 2+ | 61 (20.6%) | 9 (12.0%) | 12 (33.3%) | 18 (17.5%) | 18 (26.1%) | 1 (14.3%) | 3 (50.0%) |
| Gravidity |  |  |  |  |  |  |  |
| 1 | 99 (33.4%) | 32 (42.7%) | 11 (30.6%) | 34 (32.0%) | 17 (24.6%) | 4 (57.1%) | 1 (16.7%) |
| 2+ | 197 (66.6%) | 43 (57.3%) | 25 (69.4%) | 69 (67.0%) | 52 (75.4%) | 3 (42.9%) | 5 (83.3%) |
| Nationality (Swiss or by continent) |  |  |  |  |  |  |  |
| Swiss | 111 (37.5%) | 75 (100%) | 36 (100%) | 0 | 0 | 0 | 0 |
| Africa | 35 (11.8%) | 0 | 0 | 14 (13.6%) | 16 (23.2%) | 1 (14.3%) | 4 (66.7%) |
| North America | 3 (1.0%) | 0 | 0 | 2 (1.9%) | 1 (1.4%) | 0 | 0 |
| South America | 26 (8.8%) | 0 | 0 | 15 (14.6%) | 7 (10.1%) | 4 (57.1%) | 0 |
| Asia | 19 (6.4%) | 0 | 0 | 9 (8.7%) | 6 (8.7%) | 2 (28.6%) | 2 (33.3%) |
| Eastern Europe | 25 (8.4%) | 0 | 0 | 13 (12.6%) | 12 (17.4%) | 0 | 0 |
| Western Europe | 77 (26.0%) | 0 | 0 | 50 (48.5%) | 27 (39.1%) | 0 | 0 |
| French language acquisition |  |  |  |  |  |  |  |
| 1st | 179 (60.5%) | 45 (60.0%) | 23 (63.9%) | 60 (58.3%) | 41 (59.4%) | 6 (85.7%) | 4 (66.7%) |
| 2nd | 41 (13.9%) | 13 (17.3%) | 4 (11.1%) | 15 (14.6%) | 9 (13.0%) | 0 | 0 |
| No mention either as a 1st or 2nd language | 76 (25.7%) | 17 (22.7%) | 9 (25.0%) | 28 (27.2%) | 19 (27.5%) | 1 (14.3%) | 2 (33.3%) |
| Tariff attribute |  |  |  |  |  |  |  |
| no data | 17 (5.7%) | 5 (6.7%) | 3 (8.3%) | 5 (4.9%) | 4 (5.8%) | 0 | 0 |
| Confederate domiciled in the canton | 93 (31.4%) | 64 (85.3%) | 29 (80.6%) | 0 | 0 | 0 | 0 |
| Foreigner domiciled in the canton | 175 (59.1%) | 6 (8.0%) | 4 (11.1%) | 90 (87.4%) | 63 (91.3%) | 6 (85.7%) | 6 (100%) |
| Foreigner domiciled abroad | 1 (0.3%) | 0 | 0 | 0 | 1 (1.4%) | 0 | 0 |
| Borderer | 1 (0.3%) | 0 | 0 | 1 (1.0%) | 0 | 0 | 0 |
| International officer/mission/consulate | 8 (2.7%) | 0 | 0 | 7 (6.8%) | 1 (1.4%) | 0 | 0 |
| Domiciled in Geneva, no LAMal | 1 (0.3%) | 0 | 0 | 0 | 0 | 1 (14.3%) | 0 |
| Marital status |  |  |  |  |  |  |  |
| Single | 92 (31.1%) | 33 (44.0%) | 12 (33.3%) | 25 (24.3%) | 17 (24.6%) | 4 (57.1%) | 1 (16.7%) |
| In a relationship (concubinage) | 4 (1.4%) | 1 (1.3%) | 0 | 1 (1.0%) | 2 (2.9%) | 0 | 0 |
| Married | 186 (62.8%) | 38 (50.7%) | 21 (58.3%) | 72 (69.9%) | 48 (69.6%) | 2 (28.6%) | 5 (83.3%) |
| Separated/ divorced | 13 (4.4%) | 3 (4.0%) | 3 (8.3%) | 4 (3.9%) | 2 (2.9%) | 1 (14.3%) | 0 |
| Widow | 1 (0.3%) | 0 | 0 | 1 (1.0%) | 0 | 0 | 0 |
| Religion |  |  |  |  |  |  |  |
| No religion | 70 (23.6%) | 24 (32.0%) | 11 (30.6%) | 22 (21.4%) | 13 (18.8%) | 0 | 0 |
| Buddhism | 3 (1.0%) | 0 | 1 (2.8%) | 0 | 1 (1.4%) | 1 (14.3%) | 0 |
| Christian | 106 (35.8%) | 35 (46.7%) | 11 (30.6%) | 39 (37.9%) | 19 (27.5%) | 2 (28.6%) | 0 |
| Hinduism | 2 (0.7%) | 0 | 0 | 0 | 2 (2.9%) | 0 | 0 |
| Judaism | 1 (0.3%) | 0 | 0 | 1 (1.0%) | 0 | 0 | 0 |
| Muslim | 44 (14.9%) | 5 (6.7%) | 6 (16.7%) | 15 (14.6%) | 15 (21.7%) | 1 (14.3%) | 2 (33.3%) |
| Other | 5 (1.7%) | 2 (2.7%) | 1 (2.8%) | 1 (1.0%) | 1 (1.4%) | 0 | 0 |
| Refusal to answer | 10 (3.4%) | 2 (2.7%) | 2 (5.6%) | 5 (4.9%) | 1 (1.4%) | 0 | 0 |
| Not asked | 55 (18.6%) | 7 (9.3%) | 4 (11.1%) | 20 (19.4%) | 17 (24.6%) | 3 (42.9%) | 4 (66.7%) |
| Profession |  |  |  |  |  |  |  |
| No work | 91 (30.7%) | 10 (13.3%) | 10 (27.8%) | 29 (28.2%) | 32 (46.4%) | 4 (57.1%) | 6 (100%) |
| Student | 13 (4.4%) | 4 (5.3%) | 2 (5.6%) | 6 (5.8%) | 0 | 1 (14.3%) | 0 |
| Domestic work | 13 (4.4%) | 0 | 0 | 7 (6.8%) | 6 (8.7%) | 0 | 0 |
| Low skill work | 81 (27.4%) | 27 (36.0%) | 12 (33.3%) | 24 (23.3%) | 17 (24.6%) | 1 (14.3%) | 0 |
| High skill work | 97 (32.8%) | 34 (45.3%) | 12 (33.3%) | 37 (35.9%) | 14 (20.3%) | 0 | 0 |
| No data | 1 (0.3%) | 0 | 0 | 0 | 0 | 1 (14.3%) | 0 |
| Active smoker |  |  |  |  |  |  |  |
| Yes | 20 (6.8%) | 6 (8.0%) | 4 (11.1%) | 3 (2.9%) | 7 (10.6%) | 0 | 0 |
| No | 273 (93.2%) | 69 (92.0%) | 32 (88.9%) | 100 (97.1%) | 59 (89.4%) | 7 (100%) | 6 (100%) |
| Missing values | 3 |  |  |  | 3 |  |  |
