# Supplementary material for: Influence of women’s legal status on pregnancy outcomes and quality of care: Findings from the Pregnancy of Migrants in Switzerland (PROMISES) program
Source: PLOS Glob Public Health. 2025 Apr 21;5(4):e0004217. doi: 10.1371/journal.pgph.0004217 (PMC12011233; doi:10.1371/journal.pgph.0004217)
Supplement: S4 Table — (DOCX) [file pgph.0004217.s004.docx]

**Table 4: Obstetrical variables, by group**

| **Obstetrical variables** | **Total**  **(n= 296)** | **Swiss non precarious SNP**  **(n=75, 25.3%)** | **Swiss precarious SP (n=36, 12.2%)** | **Documented migrant non precarious DMNP (n=103, 34.8%)** | **Documented migrant precarious DMP (n=69, 23.3%)** | **Undocumented migrants UM (n=7, 2.4%)** | **Asylum seekers AS (n=6, 2.0%)** |
| --- | --- | --- | --- | --- | --- | --- | --- |
| Term |  |  |  |  |  |  |  |
| Preterm (<37 SA) | 18 (6.1%) | 5 (6.7%) | 4 (11.1%) | 5 (4.8%) | 4 (5.7%) | 0 | 0 |
| Term (37-41 6/7 SA) | 278 (93.9%) | 70 (93.3%) | 32 (88.9%) | 98 (95.1%) | 65 (94.2%) | 7 (100%) | 6 (100%) |
| Delivery type |  |  |  |  |  |  |  |
| Spontaneous vaginal birth | 178 (60.1%) | 38 (50.7%) | 21 (58.3%) | 62 (60.2%) | 47 (68.1%) | 5 (71.4%) | 5 (83.3%) |
| Instrumented vaginal birth | 41 (13.9%) | 17 (22.7%) | 5 (13.9%) | 9 (8.7%) | 9 (13.0%) | 0 | 1 (16.7%) |
| Cesarean section (C-section) | 77 (26.0%) | 20 (26.7%) | 10 (27.8%) | 32 (31.1%) | 13 (18.8%) | 2 (28.6%) | 0 |
| Induced labor |  |  |  |  |  |  |  |
| No | 123 (41.6%) | 30 (40.0%) | 14 (38.9%) | 44 (42.7%) | 28 (40.6%) | 4 (57.1%) | 3 (50.0%) |
| Yes | 132 (44.6%) | 34 (45.3%) | 16 (44.4%) | 44 (42.7%) | 33 (47.8%) | 2 (28.6%) | 3 (50.0%) |
| Cesarean section before labor | 41 (13.9%) | 11 (14.7%) | 6 (16.7%) | 15 (14.6%) | 8 (11.6%) | 1 (14.3%) | 0 |
| Past C-section |  |  |  |  |  |  |  |
| Yes | 36 (12.2%) | 8 (10.7%) | 8 (22.2%) | 11 (10.7%) | 7 (10.1%) | 1 (14.3%) | 1 (16.7%) |
| No | 260 (87.8%) | 67 (89.3%) | 28 (77.8%) | 92 (89.3%) | 62 (89.9%) | 6 (85.7%) | 5 (83.3%) |
| Single or multiple pregnancy |  |  |  |  |  |  |  |
| Multiple | 5 (1.7%) | 3 (4.0%) | 1 (2.8%) | 0 | 1 (1.4%) | 0 | 0 |
| Single | 291 (98.3%) | 72 (96.0%) | 35 (97.2%) | 103 (100%) | 68 (98.6%) | 7 (100%) | 6 (100%) |
| Anesthesia |  |  |  |  |  |  |  |
| No | 35 (11.8%) | 6 (8.0%) | 5 (13.9%) | 19 (18.4%) | 4 (5.8%) | 0 | 1 (16.7%) |
| Gas (EMONO | 8 (2.7%) | 4 (5.3%) | 0 | 1 (1.0%) | 3 (4.3%) | 0 | 0 |
| Epidural | 206 (69.6%) | 56 (74.7%) | 24 (66.7%) | 70 (68.0%) | 51 (73.9%) | 4 (57.1%) | 1 (16.7%) |
| General | 7 (2.4%) | 0 | 0 | 3 (2.9%) | 4 (5.8%) | 0 | 0 |
| Other/not described | 40 (13.5%) | 9 (12.0%) | 7 (19.4%) | 10 (9.7%) | 7 (10.1%) | 3 (42.9%) | 4 (66.7%) |
| Postpartum hemorrhage |  |  |  |  |  |  |  |
| Yes | 48 (16.2%) | 10 (13.3%) | 6 (16.7%) | 19 (18.4%) | 11 (15.9%) | 2 (28.6%) | 0 |
| No | 248 (83.8%) | 65 (86.7%) | 30 (83.3%) | 84 (81.6%) | 58 (84.1%) | 5 (71.4%) | 6 (100%) |
| Perineal tear |  |  |  |  |  |  |  |
| None | 163 (55.1%) | 37 (49.3%) | 22 (61.1%) | 60 (58.3%) | 37 (53.6%) | 4 (57.1%) | 3 (50.0%) |
| Type I | 61 (20.6%) | 12 (16.0%) | 6 (16.7%) | 25 (24.3%) | 17 (24.6%) | 0 | 1 (16.7%) |
| Type II | 69 (23.3%) | 24 (32.0%) | 8 (22.2%) | 17 (16.5%) | 15 (21.7%) | 3 (42.9%) | 2 (33.3%) |
| Type III | 3 (1.0%) | 2 (2.7%) | 0 | 1 (1.0%) | 0 | 0 | 0 |
| Episiotomy |  |  |  |  |  |  |  |
| Yes | 23 (7.8%) | 8 (10.7%) | 4 (11.1%) | 4 (3.9%) | 5 (7.2%) | 0 | 2 (33.3%) |
| No | 273 (92.2%) | 67 (89.3%) | 32 (88.9%) | 99 (96.1%) | 64 (92.8%) | 7 (100%) | 4 (66.7%) |
| Threat of preterm delivery |  |  |  |  |  |  |  |
| Yes | 8 (2.8%) | 1 (1.4%) | 1 (3.0%) | 4 (4.1%) | 2 (3.1%) | 0 | 0 |
| No | 274 (97.2%) | 73 (98.6%) | 32 (97.0%) | 94 (95.9%) | 62 (96.9%) | 7 (100%) | 6 (100%) |
| missing values | 14 | 1 | 3 | 5 | 5 |  |  |
| Gestational diabetes or diabetes |  |  |  |  |  |  |  |
| Yes | 29 (9.8%) | 6 (8.0%) | 7 (19.4%) | 10 (9.7%) | 5 (7.2%) | 1 (14.3%) | 0 |
| No | 267 (90.2%) | 69 (92.0%) | 29 (80.6%) | 93 (90.3%) | 64 (92.8%) | 6 (85.7%) | 6 (100%) |
| Gestational hypertension |  |  |  |  |  |  |  |
| Yes | 10 (3.4%) | 1 (1.3%) | 4 (11.1%) | 2 (1.9%) | 2 (2.9%) | 1 (14.3%) | 0 |
| No | 286 (96.6%) | 74 (98.7%) | 32 (88.9%) | 101 (98.1%) | 67 (97.1%) | 6 (85.7%) | 6 (100%) |
| Eclampsia or preeclampsia |  |  |  |  |  |  |  |
| Yes | 10 (3.4%) | 2 (2.7%) | 2 (5.6%) | 1 (1.0%) | 4 (5.8%) | 1 (14.3%) | 0 |
| No | 286 (96.6%) | 73 (97.3%) | 34 (94.4%) | 102 (99.0%) | 65 (94.2%) | 6 (85.7%) | 6 (100%) |
| Female genital cutting present |  |  |  |  |  |  |  |
| Yes | 12 (4.3%) | 0 | 0 | 4 (4.1%) | 4 (6.2%) | 0 | 4 (66.7%) |
| No | 270 (95.7%) | 74 (100%) | 33 (100%) | 94 (95.9%) | 60 (93.8%) | 7 (100%) | 2 (33.3%) |
| missing values | 14 | 1 | 3 | 5 | 5 |  |  |
| Newborn’s birth weight |  |  |  |  |  |  |  |
| Low (<2500 g) | 21 (7.1%) | 6 (8.0%) | 3 (8.3%) | 8 (7.8%) | 3 (4.3%) | 1 (14.3%) | 0 |
| Normal (2500-3500 g) | 258 (87.2%) | 63 (84.0%) | 31 (86.1%) | 89 (86.4%) | 63 (91.3%) | 6 (85.7%) | 6 (100%) |
| High (>4000 g) | 17 (5.7%) | 6 (8.0%) | 2 (5.6%) | 6 (5.8%) | 3 (4.3%) | 0 | 0 |
| Newborn’s hospitalization in the neonatal unit - Main diagnosis |  |  |  |  |  |  |  |
| None | 286 (96.6%) | 74 (98.7%) | 31 (86.1%) | 101 (98.1%) | 67 (97.1%) | 7 (100%) | 6 (100%) |
| Neonatal respiratory distress syndrome | 3 (1.0%) | 0 | 1 (2.8%) | 1 (1.0%) | 1 (1.4%) | 0 | 0 |
| Transient tachypnea in newborn | 1 (0.3%) | 0 | 1 (2.8%) | 0 | 0 | 0 | 0 |
| Severe obstetric asphyxia | 1 (0.3%) | 0 | 0 | 1 (1.0%) | 0 | 0 | 0 |
| Other neonatal respiratory distress | 1 (0.3%) | 0 | 1 (2.8%) | 0 | 0 | 0 | 0 |
| Low birth weight | 1 (0.3%) | 0 | 0 | 0 | 1 (1.4%) | 0 | 0 |
| Extreme prematurity | 2 (0.7%) | 1 (1.3%) | 1 (2.8%) | 0 | 0 | 0 | 0 |
| Twin pregnancy | 1 (0.3%) | 0 | 1 (2.8%) | 0 | 0 | 0 | 0 |
