# Supplementary material for: Influence of women’s legal status on pregnancy outcomes and quality of care: Findings from the Pregnancy of Migrants in Switzerland (PROMISES) program
Source: PLOS Glob Public Health. 2025 Apr 21;5(4):e0004217. doi: 10.1371/journal.pgph.0004217 (PMC12011233; doi:10.1371/journal.pgph.0004217)
Supplement: S5 Table — (DOCX) [file pgph.0004217.s005.docx]

### Table 5: Quality variables, by group

| **Quality variables** | **Total**  **(n= 296)** | **Swiss non precarious SNP**  **(n=75, 25.3%)** | **Swiss precarious SP (n=36, 12.2%)** | **Documented migrant non precarious DMNP**  **(n=103, 34.8%)** | **Documented migrant precarious DMP (n=69, 23.3%)** | **Undocumented migrants UM**  **(n=7, 2.4%)** | **Asylum seekers AS (n=6, 2.0%)** |
| --- | --- | --- | --- | --- | --- | --- | --- |
| First contact with hospital: emergency room |  |  |  |  |  |  |  |
| No | 212 (71.6%) | 50 (66.7%) | 24 (66.7%) | 76 (73.8%) | 50 (72.5%) | 7 (100%) | 5 (83.3%) |
| Yes | 84 (28.4%) | 25 (33.3%) | 12 (33.3%) | 27 (26.2%) | 19 (27.5%) | 0 | 1 (16.7%) |
| Private gynecological monitoring |  |  |  |  |  |  |  |
| No | 80 (27.0%) | 15 (20.0%) | 3 (8.3%) | 27 (26.2%) | 23 (33.3%) | 6 (85.7%) | 6 (100%) |
| Yes | 216 (73.0%) | 60 (80.0%) | 33 (91.7%) | 76 (73.8%) | 46 (66.7%) | 1 (14.3%) | 0 |
| Folic acid supplementation |  |  |  |  |  |  |  |
| No | 185 (64.2%) | 48 (64.0%) | 21 (60.0%) | 66 (66.0%) | 42 (64.6%) | 3 (42.9%) | 5 (83.3%) |
| Yes | 103 (35.8%) | 27 (36.0%) | 14 (40.0%) | 34 (34.0%) | 23 (35.4%) | 4 (57.1%) | 1 (16.7%) |
| missing values | 8 |  | 1 | 3 | 4 |  |  |
| Breastfeeding |  |  |  |  |  |  |  |
| Bottle | 49 (16.6%) | 13 (17.3%) | 4 (11.1%) | 18 (17.5%) | 14 (20.3%) | 0 | 0 |
| Breastfeeding | 247 (83.4%) | 62 (82.7%) | 32 (88.9%) | 85 (82.5%) | 55 (79.7%) | 7 (100%) | 6 (100%) |
| Admission motive |  |  |  |  |  |  |  |
| Bleeding/3rd trimester hemorrhage | 4 (1.4%) | 2 (2.7%) | 0 | 1 (1.0%) | 1 (1.4%) | 0 | 0 |
| CS | 1 (0.3%) | 0 | 0 | 1 (1.0%) | 0 | 0 | 0 |
| Decrease in fetal movements | 1 (0.3%) | 0 | 0 | 1 (1.0%) | 0 | 0 | 0 |
| Oligohydramnios /anamnios | 1 (0.3%) | 0 | 0 | 1 (1.0%) | 0 | 0 | 0 |
| Other | 1 (0.3%) | 1 (1.3%) | 0 | 0 | 0 | 0 | 0 |
| Preeclampsia (PE)/suspicion of PE | 5 (1.7%) | 0 | 2 (5.6%) | 1 (1.0%) | 2 (2.9%) | 0 | 0 |
| Pre-labor | 5 (1.7%) | 1 (1.3%) | 0 | 2 (2.0%) | 1 (1.4%) | 1 (14.3%) | 0 |
| Premature or spontaneous rupture of membrane | 41 (13.9%) | 13 (17.3%) | 6 (16.7%) | 13 (12.7%) | 7 (10.1%) | 1 (14.3%) | 1 (16.7%) |
| Spontaneous/maturation/ trigger | 228 (77.3%) | 56 (74.7%) | 26 (72.2%) | 78 (76.5%) | 58 (84.1%) | 5 (71.4%) | 5 (83.3%) |
| Suspicious of pathological CTG | 5 (1.7%) | 1 (1.3%) | 1 (2.8%) | 3 (2.9%) | 0 | 0 | 0 |
| Termination of pregnancy/ death in utero | 1 (0.3%) | 0 | 0 | 1 (1.0%) | 0 | 0 | 0 |
| Threat of preterm delivery | 2 (0.7%) | 1 (1.3%) | 1 (2.8%) | 0 | 0 | 0 | 0 |
| Missing values | 1 |  |  | 1 |  |  |  |
