# Supplementary material for: Influence of women’s legal status on pregnancy outcomes and quality of care: Findings from the Pregnancy of Migrants in Switzerland (PROMISES) program
Source: PLOS Glob Public Health. 2025 Apr 21;5(4):e0004217. doi: 10.1371/journal.pgph.0004217 (PMC12011233; doi:10.1371/journal.pgph.0004217)
Supplement: S6 Table — (DOCX) [file pgph.0004217.s006.docx]

### Table 6: Quality variables for women with no private care

| **Quality variables** | **Total**  **(n=81)** | **Swiss non precarious SNP (n=15, 18.5%)** | **Swiss precarious SP (n=3, 3.7%)** | **Documented migrant non-precarious DMNP**  **(n=27, 33.3%)** | **Documented migrant precarious DMP (n=24, 29.6%)** | **Undocumented migrants UM**  **(n=6, 7.4%)** | **Asylum seekers AS**  **(n=6, 7.4%)** |
| --- | --- | --- | --- | --- | --- | --- | --- |
| Timely ultrasound |  |  |  |  |  |  |  |
| Yes | 59 (73.8%) | 13 (86.7%) | 3 (100%) | 19 (70.4%) | 18 (78.3%) | 4 (66.7%) | 2 (33.3%) |
| No | 21 (26.2%) | 2 (13.3%) | 0 | 8 (29.6%) | 5 (21.7%) | 2 (33.3%) | 4 (66.7%) |
| Appropriate time for first contact |  |  |  |  |  |  |  |
| Yes | 4 (66.7%) | 0 | 0 | 0 | 0 | 4 (66.7%) | 0 |
| No | 2 (33.3%) | 0 | 0 | 0 | 0 | 2 (33.3%) | 0 |
| Missing values | 75 | 15 | 3 | 27 | 24 |  | 6 |
