# Supplementary material for: Influence of women’s legal status on pregnancy outcomes and quality of care: Findings from the Pregnancy of Migrants in Switzerland (PROMISES) program
Source: PLOS Glob Public Health. 2025 Apr 21;5(4):e0004217. doi: 10.1371/journal.pgph.0004217 (PMC12011233; doi:10.1371/journal.pgph.0004217)
Supplement: S7 Table — (DOCX) [file pgph.0004217.s007.docx]

### Table 7: Socio-demographic variables comparing precarious vs. non-precarious documented migrant women

| **Socio-demographic variables** | **Documented migrant non-precarious DMNP**  **(n=103, 59.9%)** | **Documented migrant precarious DMP**  **(n=69, 40.1%)** | **p-value (Mann-Whitney/chi^2^)** |
| --- | --- | --- | --- |
| Age of patient | 32 (29 - 36) | 32 (27 - 37) | 0.570 |
| Parity |  |  | 0.383 |
| 0 | 47 (45.6%) | 26 (37.7%) |  |
| 1 | 38 (36.9%) | 25 (36.2%) |  |
| 2+ | 18 (17.5%) | 18 (26.1%) |  |
| Gravidity |  |  | 0.313¹ |
| 1 | 34 (33.0%) | 17 (24.6%) |  |
| 2+ | 69 (67.0%) | 52 (75.4%) |  |
| Tariff attribute |  |  | 0.353¹ |
| no data | 5 (4.9%) | 4 (5.8%) |  |
| Foreigner domiciled in the canton | 1 (1.0%) | 0 |  |
| Foreigner domiciled abroad | 89 (86.4%) | 63 (91.3%) |  |
| Borderer | 0 | 1 (1.4%) |  |
| International officer/mission/consulate | 1 (1.0%) | 0 |  |
| Domiciled in Geneva, no LAMal | 7 (6.8%) | 1 (1.4%) |  |
| French acquisition |  |  | 0.961 |
| 1st | 60 (58.3%) | 41 (59.4%) |  |
| 2nd | 15 (14.6%) | 9 (13.0%) |  |
| no mention | 28 (27.2%) | 19 (27.5%) |  |
| Marital status |  |  | 0.909¹ |
| 0 | 25 (24.3%) | 17 (24.6%) |  |
| 1 | 1 (1.0%) | 2 (2.9%) |  |
| 2 | 72 (69.9%) | 48 (69.6%) |  |
| 3 | 4 (3.9%) | 2 (2.9%) |  |
| 4 | 1 (1.0%) | 0 |  |
| Religion |  |  | 0.214¹ |
| No religion | 22 (21.4%) | 13 (18.8%) |  |
| Buddhism | 0 | 1 (1.4%) |  |
| Christian | 40 (38.8%) | 19 (27.5%) |  |
| Hinduism | 0 | 2 (2.9%) |  |
| Judaism | 1 (1.0%) | 0 |  |
| Muslim | 15 (14.6%) | 15 (21.7%) |  |
| Other | 1 (1.0%) | 1 (1.4%) |  |
| Refusal to answer | 5 (4.9%) | 1 (1.4%) |  |
| Not asked | 19 (18.4%) | 17 (24.6%) |  |
| Profession |  |  | 0.015¹ |
| No work | 29 (28.2%) | 32 (46.4%) |  |
| Student | 6 (5.8%) | 0 |  |
| Domestic work | 7 (6.8%) | 6 (8.7%) |  |
| Low skill work | 23 (22.3%) | 17 (24.6%) |  |
| High skill work | 38 (36.9%) | 14 (20.3%) |  |
| Active smoker |  |  | 0.049¹ |
| Yes | 100 (97.1%) | 59 (89.4%) |  |
| No | 3 (2.9%) | 7 (10.6%) |  |
| missing values |  | 3 |  |

¹Fisher’s exact
