# Supplementary material for: Influence of women’s legal status on pregnancy outcomes and quality of care: Findings from the Pregnancy of Migrants in Switzerland (PROMISES) program
Source: PLOS Glob Public Health. 2025 Apr 21;5(4):e0004217. doi: 10.1371/journal.pgph.0004217 (PMC12011233; doi:10.1371/journal.pgph.0004217)
Supplement: S8 Table — (DOCX) [file pgph.0004217.s008.docx]

**Table 8: Obstetrical variables comparing precarious vs. non-precarious documented migrant women**

| **Obstetrical variables** | **Documented migrant non-precarious DMNP**  **(n=103, 59.9%)** | **Documented migrant precarious DMP**  **(n=69, 40.1%)** | **p-value (Mann-Whitney/chi^2^)** |
| --- | --- | --- | --- |
| Term |  |  | 0.863¹ |
| Preterm (<37 SA) | 5 (4.8%) | 4 (5.7%) |  |
| Term (37-41 6/7 SA) | 98 (95.1%) | 65 (94.2%) |  |
| Delivery type |  |  | 0.192 |
| Spontaneous vaginal birth | 61 (59.2%) | 47 (68.1%) |  |
| Instrumented vaginal birth | 10 (9.7%) | 9 (13.0%) |  |
| Cesarean section | 32 (31.1%) | 13 (18.8%) |  |
| Induced labor |  |  | 0.700 |
| No | 45 (43.7%) | 28 (40.6%) |  |
| Yes | 43 (41.7%) | 33 (47.8%) |  |
| Cesarean section before labor | 15 (14.6%) | 8 (11.6%) |  |
| Past CS |  |  | 0.999 |
| No | 92 (89.3%) | 62 (89.9%) |  |
| Yes | 11 (10.7%) | 7 (10.1%) |  |
| Single or multiple pregnancy |  |  | 0.401¹ |
| No | 103 (100%) | 68 (98.6%) |  |
| Yes | 0 | 1 (1.4%) |  |
| Anesthesia |  |  | 0.097¹ |
| No | 18 (17.5%) | 4 (5.8%) |  |
| Gaz (EMONO) | 1 (1.0%) | 3 (4.3%) |  |
| Epidural | 71 (68.9%) | 51 (73.9%) |  |
| General | 3 (2.9%) | 4 (5.8%) |  |
| Other/not described | 10 (9.7%) | 7 (10.1%) |  |
| Postpartum hemorrhage |  |  | 0.826 |
| No | 84 (81.6%) | 58 (84.1%) |  |
| Yes | 19 (18.4%) | 11 (15.9%) |  |
| Perineal tear |  |  | 0.852¹ |
| None | 60 (58.3%) | 37 (53.6%) |  |
| Type I | 24 (23.3%) | 17 (24.6%) |  |
| Type II | 18 (17.5%) | 15 (21.7%) |  |
| Type III | 1 (1.0%) | 0 |  |
| Episiotomy |  |  | 0.525¹ |
| Yes | 98 (95.1%) | 64 (92.8%) |  |
| No | 5 (4.9%) | 5 (7.2%) |  |
| Threat of preterm delivery |  |  | 0.999¹ |
| No | 94 (95.9%) | 62 (96.9%) |  |
| Yes | 4 (4.1%) | 2 (3.1%) |  |
| missing values | 5 | 5 |  |
| Gestational diabetes or diabetes |  |  | 0.775 |
| No | 93 (90.3%) | 64 (92.8%) |  |
| Yes | 10 (9.7%) | 5 (7.2%) |  |
| Gestational hypertension |  |  | 0.999¹ |
| No | 101 (98.1%) | 67 (97.1%) |  |
| Yes | 2 (1.9%) | 2 (2.9%) |  |
| Eclampsia or preeclampsia |  |  | 0.159¹ |
| No | 102 (99.0%) | 65 (94.2%) |  |
| Yes | 1 (1.0%) | 4 (5.8%) |  |
| Female genital cutting |  |  | 0.713¹ |
| No | 94 (95.9%) | 60 (93.8%) |  |
| Yes | 4 (4.1%) | 4 (6.2%) |  |
| missing values | 5 | 5 |  |
| Newborn’s birth weight |  |  | 0.711¹ |
| Low (<2500 g) | 8 (7.8%) | 3 (4.3%) |  |
| Normal (2500-3500 g) | 89 (86.4%) | 63 (91.3%) |  |
| High (4000 g) | 6 (5.8%) | 3 (4.3%) |  |
| Newborn’s hospitalization in the neonatal unit - Main diagnosis |  |  | 0.701¹ |
| none | 101 (98.1%) | 67 (97.1%) |  |
| neonatal respiratory distress syndrome | 1 (1.0%) | 1 (1.4%) |  |
| severe obstetric asphyxia | 1 (1.0%) | 0 |  |
| low birth weight | 0 | 1 (1.4%) |  |

¹Fisher’s exact
