# Supplementary material for: Influence of women’s legal status on pregnancy outcomes and quality of care: Findings from the Pregnancy of Migrants in Switzerland (PROMISES) program
Source: PLOS Glob Public Health. 2025 Apr 21;5(4):e0004217. doi: 10.1371/journal.pgph.0004217 (PMC12011233; doi:10.1371/journal.pgph.0004217)
Supplement: S9 Table — (DOCX) [file pgph.0004217.s009.docx]

**Table 9: Quality variables comparing precarious vs. non-precarious documented migrant women**

| **Quality variables** | **Documented migrant non-precarious (n=103, 59.9%)** | **Documented migrant precarious**  **(n=69, 40.1%)** | **p-value (Mann-Whitney/chi^2^)** |
| --- | --- | --- | --- |
| First contact with hospital: emergency room |  |  | 0.874 |
| No | 77 (74.8%) | 50 (72.5%) |  |
| Yes | 26 (25.2%) | 19 (27.5%) |  |
| Private gynecological monitoring |  |  | 0.403 |
| No | 27 (26.2%) | 23 (33.3%) |  |
| Yes | 76 (73.8%) | 46 (66.7%) |  |
| Folic acid supplementation |  |  | 0.882 |
| No | 67 (67.0%) | 42 (64.6%) |  |
| Yes | 33 (33.0%) | 23 (35.4%) |  |
| missing values | 3 | 4 |  |
| Breastfeeding |  |  | 0.791 |
| No | 18 (17.5%) | 14 (20.3%) |  |
| Yes | 85 (82.5%) | 55 (79.7%) |  |
| Admission motive |  |  | 0.866¹ |
| bleeding/3rd trimester hemorrhage | 1 (1.0%) | 1 (1.4%) |  |
| CS | 1 (1.0%) | 0 |  |
| decrease fetal movements | 1 (1.0%) | 0 |  |
| oligohydramnios /anamnios | 1 (1.0%) | 0 |  |
| preeclampsia/suspicion of PE | 1 (1.0%) | 2 (2.9%) |  |
| pre-labor | 2 (2.0%) | 1 (1.4%) |  |
| premature or spontaneous rupture of membrane | 14 (13.7%) | 7 (10.1%) |  |
| spontaneous/maturation/trigger | 77 (75.5%) | 58 (84.1%) |  |
| suspicion of pathological cardiotocogram | 3 (2.9%) | 0 |  |
| termination of pregnancy/death in utero | 1 (1.0%) | 0 |  |
| missing values | 1 |  |  |
| Timely ultrasound |  |  | 0.756 |
| No | 8 (29.6%) | 5 (21.7%) |  |
| Yes | 19 (70.4%) | 18 (78.3%) |  |
| missing values | 76 | 46 |  |
| Appropriate time for first contact |  |  | - |
| missing values | 103 | 69 |  |

¹Fisher’s exact
