# Supplementary material for: Influence of women’s legal status on pregnancy outcomes and quality of care: Findings from the Pregnancy of Migrants in Switzerland (PROMISES) program
Source: PLOS Glob Public Health. 2025 Apr 21;5(4):e0004217. doi: 10.1371/journal.pgph.0004217 (PMC12011233; doi:10.1371/journal.pgph.0004217)
Supplement: S10 Table — (DOCX) [file pgph.0004217.s010.docx]

### Table 10: Sociodemographic variables for precarious women, Swiss vs. documented migrants

| **Sociodemographic variables** | **Swiss precarious SP**  **(n=36, 34.3%)** | **Documented migrant precarious DMP**  **(n=69, 65.7%)** | **p-value (Mann-Whitney/chi^2^)** |
| --- | --- | --- | --- |
| Age of patient |  |  | 0.779 |
|  | 31 (29 - 34.2) | 32 (27 - 37) |  |
| Parity |  |  | 0.414 |
| 0 | 16 (44.4%) | 26 (37.7%) |  |
| 1 | 8 (22.2%) | 25 (36.2%) |  |
| 2+ | 12 (33.3%) | 18 (26.1%) |  |
| Gravidity |  |  | 0.676¹ |
| 1 | 11 (30.6%) | 17 (24.6%) |  |
| 2+ | 25 (69.4%) | 52 (75.4%) |  |
| Tariff attribute |  |  | < 0.001¹ |
| no data | 3 (8.3%) | 4 (5.8%) |  |
| Confederate domiciled in the canton | 29 (80.6%) | 0 |  |
| Foreigner domiciled in the canton | 4 (11.1%) | 63 (91.3%) |  |
| Foreigner domiciled abroad | 0 | 1 (1.4%) |  |
| International officer/mission/consulate | 0 | 1 (1.4%) |  |
| French |  |  | 0.956¹ |
| 1st | 23 (63.9%) | 41 (59.4%) |  |
| 2nd | 4 (11.1%) | 9 (13.0%) |  |
| no mention | 9 (25.0%) | 19 (27.5%) |  |
| Marital status |  |  | 0.337¹ |
| Single | 12 (33.3%) | 17 (24.6%) |  |
| In a relationship (concubinage) | 0 | 2 (2.9%) |  |
| Married | 21 (58.3%) | 48 (69.6%) |  |
| Separated/divorced | 3 (8.3%) | 2 (2.9%) |  |
| Religion |  |  | 0.382¹ |
| No religion | 11 (30.6%) | 13 (18.8%) |  |
| Buddhism | 1 (2.8%) | 1 (1.4%) |  |
| Christian | 11 (30.6%) | 19 (27.5%) |  |
| Hinduism | 0 | 2 (2.9%) |  |
| Muslim | 6 (16.7%) | 15 (21.7%) |  |
| Other | 1 (2.8%) | 1 (1.4%) |  |
| Refusal to answer | 2 (5.6%) | 1 (1.4%) |  |
| Not asked | 4 (11.1%) | 17 (24.6%) |  |
| Profession |  |  | 0.020¹ |
| No work | 10 (27.8%) | 32 (46.4%) |  |
| Student | 2 (5.6%) | 0 |  |
| Domestic work | 0 | 6 (8.7%) |  |
| Low skill work | 12 (33.3%) | 17 (24.6%) |  |
| High skill work | 12 (33.3%) | 14 (20.3%) |  |
| Active smoker |  |  | 0.999¹ |
| No | 32 (88.9%) | 59 (89.4%) |  |
| Yes | 4 (11.1%) | 7 (10.6%) |  |
| missing values |  | 3 |  |

¹Fisher’s exact
