# Supplementary material for: Influence of women’s legal status on pregnancy outcomes and quality of care: Findings from the Pregnancy of Migrants in Switzerland (PROMISES) program
Source: PLOS Glob Public Health. 2025 Apr 21;5(4):e0004217. doi: 10.1371/journal.pgph.0004217 (PMC12011233; doi:10.1371/journal.pgph.0004217)
Supplement: S11 Table — (DOCX) [file pgph.0004217.s011.docx]

**Table 11: Obstetrical variables for precarious women, Swiss vs. documented migrants**

| **Obstetrical variables** | **Swiss precarious SP**  **(n=36, 34.3%)** | **Documented migrant precarious DMP**  **(n=69, 65.7%)** | **p-value (Mann-Whitney/chi2)** |
| --- | --- | --- | --- |
| Term |  |  | 0.249¹ |
| Preterm (<37 SA) | 4 (11.1%) | 4 (5.7%) |  |
| Term (37-41 6/7 SA) | 32 (88.9%) | 65 (94.2%) |  |
| Delivery type |  |  | 0.489¹ |
| Spontaneous vaginal birth | 21 (58.3%) | 47 (68.1%) |  |
| Instrumented vaginal birth | 5 (13.9%) | 9 (13.0%) |  |
| Cesarean section | 10 (27.8%) | 13 (18.8%) |  |
| Induced labor |  |  | 0.779¹ |
| No | 14 (38.9%) | 28 (40.6%) |  |
| Yes | 16 (44.4%) | 33 (47.8%) |  |
| Cesarean section before labor | 6 (16.7%) | 8 (11.6%) |  |
| Past CS |  |  | 0.166 |
| No | 28 (77.8%) | 62 (89.9%) |  |
| Yes | 8 (22.2%) | 7 (10.1%) |  |
| Single or multiple pregnancy |  |  | 0.999¹ |
| No | 35 (97.2%) | 68 (98.6%) |  |
| Yes | 1 (2.8%) | 1 (1.4%) |  |
| Anesthesia |  |  | 0.148¹ |
| No | 5 (13.9%) | 4 (5.8%) |  |
| Gaz (EMONO) | 0 | 3 (4.3%) |  |
| Epidural | 24 (66.7%) | 51 (73.9%) |  |
| General | 0 | 4 (5.8%) |  |
| Other/not described | 7 (19.4%) | 7 (10.1%) |  |
| Postpartum hemorrhage |  |  | 0.999 |
| No | 30 (83.3%) | 58 (84.1%) |  |
| Yes | 6 (16.7%) | 11 (15.9%) |  |
| Perineal tear |  |  | 0.630 |
| None | 22 (61.1%) | 37 (53.6%) |  |
| Type I | 6 (16.7%) | 17 (24.6%) |  |
| Type II | 8 (22.2%) | 15 (21.7%) |  |
| Episiotomy |  |  | 0.489¹ |
| No | 32 (88.9%) | 64 (92.8%) |  |
| Yes | 4 (11.1%) | 5 (7.2%) |  |
| Threat of preterm delivery |  |  | 0.999¹ |
| No | 32 (97.0%) | 62 (96.9%) |  |
| Yes | 1 (3.0%) | 2 (3.1%) |  |
| missing values | 3 | 5 |  |
| Gestational diabetes or diabetes |  |  | 0.102¹ |
| No | 29 (80.6%) | 64 (92.8%) |  |
| Yes | 7 (19.4%) | 5 (7.2%) |  |
| Gestational hypertension |  |  | 0.178¹ |
| No | 32 (88.9%) | 67 (97.1%) |  |
| Yes | 4 (11.1%) | 2 (2.9%) |  |
| Eclampsia or preeclampsia |  |  | 0.999¹ |
| No | 34 (94.4%) | 65 (94.2%) |  |
| Yes | 2 (5.6%) | 4 (5.8%) |  |
| Female genital mutilation |  |  | 0.296¹ |
| No | 33 (100%) | 60 (93.8%) |  |
| Yes | 0 | 4 (6.2%) |  |
| missing values | 3 | 5 |  |
| Newborn’s birth weight |  |  | 0.684¹ |
| Low (<2500 g) | 3 (8.3%) | 3 (4.3%) |  |
| Normal (2500-3500 g) | 31 (86.1%) | 63 (91.3%) |  |
| High (>3500 g) | 2 (5.6%) | 3 (4.3%) |  |
| Newborn’s hospitalization in the neonatal unit - Main diagnosis |  |  | 0.045¹ |
| none | 31 (86.1%) | 67 (97.1%) |  |
| neonatal respiratory distress syndrome | 1 (2.8%) | 1 (1.4%) |  |
| transient tachypnea in newborn | 1 (2.8%) | 0 |  |
| other neonatal respiratory distress | 1 (2.8%) | 0 |  |
| low birth weight | 0 | 1 (1.4%) |  |
| extreme prematurity | 1 (2.8%) | 0 |  |
| twin pregnancy | 1 (2.8%) | 0 |  |

¹Fisher’s exact
