# Supplementary material for: Influence of women’s legal status on pregnancy outcomes and quality of care: Findings from the Pregnancy of Migrants in Switzerland (PROMISES) program
Source: PLOS Glob Public Health. 2025 Apr 21;5(4):e0004217. doi: 10.1371/journal.pgph.0004217 (PMC12011233; doi:10.1371/journal.pgph.0004217)
Supplement: S12 Table — (DOCX) [file pgph.0004217.s012.docx]

**Table 12: Quality variables among precarious women, Swiss vs. documented migrants**

| **Quality variables** | **Swiss precarious SP**  **(n=36, 34.3%)** | **Documented migrant precarious DMP**  **(n=69, 65.7%)** | **p-value (Mann-Whitney/chi2)** |
| --- | --- | --- | --- |
| First contact with hospital: emergency room |  |  | 0.694 |
| No | 24 (66.7%) | 50 (72.5%) |  |
| Yes | 12 (33.3%) | 19 (27.5%) |  |
| Private gynecological monitoring |  |  | 0.010 |
| No | 3 (8.3%) | 23 (33.3%) |  |
| Yes | 33 (91.7%) | 46 (66.7%) |  |
| Folic acid supplementation |  |  | 0.811 |
| No | 21 (60.0%) | 42 (64.6%) |  |
| Yes | 14 (40.0%) | 23 (35.4%) |  |
| missing values | 1 | 4 |  |
| Breastfeeding |  |  | 0.362 |
| No | 4 (11.1%) | 14 (20.3%) |  |
| Yes | 32 (88.9%) | 55 (79.7%) |  |
| Intention to breastfeed |  |  | 0.418¹ |
| No | 1 (2.8%) | 6 (8.7%) |  |
| Yes | 35 (97.2%) | 63 (91.3%) |  |
| Admission motive |  |  | 0.301¹ |
| bleeding/3rd trimester hemorrhage | 0 | 1 (1.4%) |  |
| preeclampsia/suspicion of PE | 2 (5.6%) | 2 (2.9%) |  |
| pre-labour | 0 | 1 (1.4%) |  |
| premature or spontaneous rupture of membrane | 6 (16.7%) | 7 (10.1%) |  |
| spontaneous/maturation/trigger | 26 (72.2%) | 58 (84.1%) |  |
| suspicion of pathological cardiotocogram | 1 (2.8%) | 0 |  |
| threat of preterm delivery | 1 (2.8%) | 0 |  |
| Timely US |  |  | 0.999¹ |
| No | 0 | 5 (21.7%) |  |
| Yes | 3 (100%) | 18 (78.3%) |  |
| missing values | 33 | 46 |  |
| Appropriate time for first contact |  |  | - |
| missing values | 36 | 69 |  |

¹Fisher’s exact
