# Supplementary material for: Influence of women’s legal status on pregnancy outcomes and quality of care: Findings from the Pregnancy of Migrants in Switzerland (PROMISES) program
Source: PLOS Glob Public Health. 2025 Apr 21;5(4):e0004217. doi: 10.1371/journal.pgph.0004217 (PMC12011233; doi:10.1371/journal.pgph.0004217)

PLOS Global Public Health

Influence of women’s legal status on pregnancy outcomes and quality of care: Findings from the Pregnancy of Migrants in Switzerland (PROMISES) program

--Manuscript Draft--

**Manuscript Number:**	PGPH-D-24-01793
**Article Type:**	Research Article
**Full Title:**	Influence of women’s legal status on pregnancy outcomes and quality of care: Findings from the Pregnancy of Migrants in Switzerland (PROMISES) program
**Short Title:**	Legal status, pregnancy outcomes, and quality of care in Geneva, Switzerland
**Corresponding Author:**	Anne-Caroline Benski, M.D.<br>HUG: Hopitaux Universitaires Geneve<br>Geneva, SWITZERLAND
**Order of Authors:**	Eugénie de Weck
	Clara Levy Noble
	Jessica Sormani
	Monique Lamuela Naulin
	Cyril Jaksic
	Sara Arsever
	B. Martinez de Tejada
	Nicole C. Schmidt
	Anya Levy Guyer
	Anne-Caroline Benski
**Keywords:**	Pregnancy; migrants; asylum seekers; economic precarity; high-income country
**Abstract:**	Background: In 2020, an estimated 281 million people lived in a country other than their country of birth. In Geneva, Switzerland, people born in other countries constitute 40.2% of the population. We aimed to describe the pregnant women delivering at the University Hospitals of Geneva (HUG) maternity department sought to identify associations between legal status, migration status, and economic precarity with quality of obstetrical care and health outcomes.<br><br>Methods: We performed a retrospective cross-sectional exploratory study including all women who delivered in HUG’s maternity department in May 2019 (n=339). The group was sub-divided in three ways: by migration status (either Swiss (S) or migrant (M)); by legal status (documented (D), undocumented (U), or asylum seeker (AS)); and economic precariousness (precarious (P) or non-precarious (NP)). The analysis compared the quality of care received and health outcomes across six sub-groups: Swiss non-precarious women (SNP, 25.3%), Swiss precarious women (SP, 12.2%), documented migrant non-precarious women (DMNP, 34.8%), documented migrants precarious (DMP, 23.3%), undocumented migrants (UM, 2.4%) and asylum seekers (AS, 2.0%).<br><br>Results: Precarious patients represented 35.5% of the sample. Economic precarity was more highly associated with poor health outcomes than legal or migration status.<br><br>Conclusions: The findings from this exploratory study suggest that, where a program exists to reach pregnant undocumented migrants, a pregnant woman’s economic status is also very important in determining her experience with the healthcare system during pregnancy and delivery. More than a third of pregnant women delivering at HUG are in a situation of vulnerability, whether economic or legal. However, due to small sample sizes this study had few statistically significant results. However, it clearly highlights the need for further research into how best to address various vulnerability factors during pregnancy.

Powered by Editorial Manager® and ProduXion Manager® from Aries Systems Corporation

Additional Information:	
Question	Response
**Financial Disclosure**<br><br>Enter a financial disclosure statement that describes the sources of funding for the work included in this submission and the role the funder(s) played. This includes grants and any commercial funding of the work or authors.<br><br>This statement will be typeset if the manuscript is accepted for publication.<br><br>*Please review the submission guidelines and the instructions link below for detailed requirements and guidance.*	The authors acknowledge the "Fondation Privée des HUG" for supporting our research.
**Competing Interests**<br><br>On behalf of all authors, disclose any competing interests that could be perceived to bias this work.<br><br>This statement will be typeset if the manuscript is accepted for publication.<br><br>*Please review the instructions link below and PLOS Global Public Health's competing interests policy to determine what information must be disclosed at submission.*	No competing interests to report
**Data Availability**<br><br>Before publication, Authors are required to make fully available and without restriction all data underlying their findings. Please see our PLOS Data Policy page for detailed information on this policy.<br><br>A **Data Availability Statement**, detailing	Data are available from the corresponding author

Powered by Editorial Manager® and ProduXion Manager® from Aries Systems Corporation

where the data can be accessed, is required at first submission. Insert your Data Availability Statement in the box below.<br><br>Please see the data reporting section of our submission guidelines for instructions on what you need to include in your Data Availability Statement.<br><br>This statement will be typeset if the manuscript is accepted for publication.<br><br>*PLOS allows rare exemptions to address legal and ethical concerns. If you have legal or ethical restrictions, please use the box below to detail these in full sentences for the Journal team to consider.*	

Powered by Editorial Manager® and ProduXion Manager® from Aries Systems Corporation

Manuscript

Click here to access/download;Manuscript;Manuscript10.4.2024.docx

FULL TITLE:

Influence of women's legal status on pregnancy outcomes and quality of care: Findings from the Pregnancy of Migrants in Switzerland (PROMISES) program

SHORT TITLE:

Legal status, pregnancy outcomes, and quality of care in Geneva, Switzerland

AUTHORS:

Eugénie de Weck* 0009-0006-1944-1801 (1)
Clara Noble* (1)
Jessica Sormani 0000-0001-7111-3975 (2)
Monique Lamuela Naulin (3)
Cyril Jaksic 0000-0001-5835-3480 (4)
Sara Arsever 0000-0002-3611-8321 (5)
B. Martinez de Tejada 0000-0001-5737-3586 (1,3)
Nicole C. Schmidt 0000-0001-6803-5179 (3,6)
Anya Levy Guyer 0000-0002-7948-5287 (7)
Anne-Caroline Benski[ǂ] 0000-0002-1184-882X (1,3)

AUTHOR AFFILIATIONS:

(1) University of Geneva, Faculty of Medicine, Switzerland
(2) University of Applied Sciences and Arts Western Switzerland (HES-SO), Geneva School of Health Sciences Switzerland
(3) University Hospitals of Geneva, Department of Women, Child and Adolescent, Switzerland
(4) University Hospitals of Geneva, Clinical Epidemiology Department, Switzerland
(5) University Hospitals of Geneva, Primary Care Division, Switzerland
(6) Catholic University of Applied Science, Munich, Germany
(7) Independent Consultant, Boston, MA, USA

*Eugénie de Weck and Clara Noble contributed equally to this work
[ǂ]Corresponding author: A.-C. Benski anne-caroline.benski@hcuge.ch

AUTHORS' CONTRIBUTIONS:

Conceptualization and study design, E.D.W., C.N, C.B, .; methodology; validation, all authors.; formal analysis, E.D.W, C.N, J.S, C.J., writing—original draft preparation, E.D.W and C.N; writing of the paper, E.D.W, C.N., C.B. ; revision of the paper M.L.N., S.A., N.S., B.M.T., N.S, and final revision of the paper A.L.G.; supervision, C.B.
All authors have read and agreed to the final submitted version of the manuscript.

Influence of women's legal status on pregnancy outcomes and quality of care: Findings from the Pregnancy of Migrants in Switzerland (PROMISES) program

Abstract

Background: In 2020, an estimated 281 million people lived in a country other than their country of birth. In Geneva, Switzerland, people born in other countries constitute 40.2% of the population. We aimed to describe the population of pregnant women delivering at the University Hospitals of Geneva (HUG) maternity department and to identify associations between legal status, migration status, and economic precarity with quality of obstetrical care and health outcomes.

Methods: We performed an exploratory cross-sectional retrospective study including all women who delivered in HUG's maternity department in May 2019 (n=339). The group was sub-divided in three ways: by migration status (Swiss (S) or migrant (M)); by legal status (documented (D), undocumented (U), or asylum seeker (AS)); and economic precariousness (precarious (P) or non-precarious (NP)). The analysis compared the quality of care received and health outcomes across six sub-groups: Swiss non-precarious women (SNP, 25.3%), Swiss precarious women (SP, 12.2%), documented migrant non-precarious women (DMNP, 34.8%), documented migrant precarious women (DMP, 23.3%), undocumented migrants (UM, 2.4%), and asylum seekers (AS, 2.0%).

Results: Precarious patients represented 35.5% of the sample. Economic precarity was more highly associated with poor health outcomes than legal or migration status.

Conclusions: The findings from this exploratory study suggest that, where a program exists to reach pregnant undocumented migrants, a pregnant woman's economic status is also very important in determining her experience with the healthcare system during pregnancy and delivery. More than one third of pregnant women delivering at HUG are in a situation of vulnerability, whether economic or legal. This study had few statistically significant results due to small sample sizes. However, it clearly highlights the need for further research into how best to address various vulnerability factors during pregnancy.

Keywords: Pregnancy, migrants, asylum seeker, economic precarity, high-income country

Background

An estimated 281 million people were living in a country other than their country of birth in 2020 and almost one-third (87 million) of the world's international migrants were living in Europe [1]. Among these, 48% were women, almost half of whom were of reproductive age [2]. People migrate to other countries for diverse reasons, including economic and educational opportunities, family connections, or fleeing natural disaster or conflict [1].

In 2020 in Switzerland, 38% of the population was born in another country [3]. This number is equally divided between males and females, putting Switzerland in line with data for Europe in general, in which 51.6% of migrants are women [2]. In Geneva canton, the statistical office (Office cantonal de la statistique, OCSTAT) reported that 60.9% of the population had foreign origins [4].

The migrant population in Switzerland includes people with and without valid residency permits. “Documented” migrants include all persons of foreign origin living in the country on a legal basis (working, studying, etc.) and asylum seekers. Asylum seekers are settled by the Swiss government throughout the country. By the end of 2020, there were 377 asylum seekers in Geneva [5]. "Undocumented” migrants are those without valid residency permits; these migrants without a legal basis for being in the country are also known as “sans-papiers” (“without papers” in English). The sans-papiers either arrive in Switzerland with a tourist visa and then do not leave or enter without a legal permit. An estimated 2.7% of the total population of Geneva—approximately 13,000 people—is undocumented [6].

Migrants in Switzerland, as elsewhere, may experience social, economic, health, and other forms of “vulnerability” [7]. Vulnerable persons are not intrinsically fragile, but they are less protected by collective social structures [8]. Economic insecurity in particular, also known as “precarity”, is known to have an impact on health outcomes, in part because it is linked with delayed or no access to healthcare [8].

One source of vulnerability for migrants relates to access to health coverage. In Switzerland, the federal law on health insurance (L’assurance maladie obligatoire, or LAMal) mandates that every person living in the country for more than three months must have health insurance. Asylum seekers in Geneva receive health insurance with the broker Swiss Risk & Care [9]. However, undocumented migrants may not be able to purchase health insurance.

Recent data suggest that 90% of undocumented migrants in Geneva canton do not possess any form of health insurance (representing 5-8% of city residents and 2-3% of the canton population) [10].

In addition to the risks inherent in lacking legal status, health coverage, and social security, migrants' individual characteristics can also contribute to vulnerability. Health literacy is known to be a major obstacle for migrant women during pregnancy. There is strong evidence that vulnerability among migrant women affects their access to health care and support during pregnancy. Access to antenatal care for undocumented migrants has been described as sporadic [11]. Being a refugee or migrant is considered a risk factor for limited or delayed access to health care; migrant status may also be associated with other risk factors, including lower socioeconomic status, higher burden of disease in the country of origin, or language difficulties [12-14]. Vulnerable pregnant migrants have higher rates of adverse obstetrical and perinatal outcomes [13], including higher rates of mortality and morbidity [15,16] and more frequent mental health issues [13,17-18]. Delays in accessing care are frequent, including postponing antenatal care to prioritize more urgent situations [12]. Lack of patient follow-up after delivery is another major issue for migrant women, who are susceptible to chronic illnesses and other health problems [19-21].

Studies investigating social determinants that affect the health of pregnant migrants in Switzerland have identified several factors, including: financial constraints, language barriers, perceived or documented discrimination, feelings of embarrassment during obstetric visits, stress due to legal status, and lack of family support networks [22-23]. Delayed and sporadic patterns of access to antenatal care have also been recorded in Geneva's undocumented women [24-25].

The number of migrant women of childbearing age living in Switzerland is growing [26]. Responding to the diverse health care needs of pregnant women with migrant backgrounds presents a complex challenge. WHO technical guidance on the health care needs of migrant women recommends: increasing migrants' and health professionals' awareness of programs that address migrant patients' needs, expanding access to translation services, and addressing affordability [27].

The University Hospitals of Geneva (HUG) is the leading health care provider in the Geneva canton. The HUG maternity department is the only public maternity hospital in the canton. In 2019, 83% of births (4248 out of 5111)

in the Geneva canton occurred there [28-29]. HUG has already adapted its services to better fit vulnerable migrant women. The CAMSCO program (Ambulatory Mobile Community Care Consultation) was established in 1997 to foster access to health care for vulnerable populations. CAMSCO provides pregnant undocumented migrant women with access to health care provided by midwives specialized in migrant health. Furthermore, interpreters are available at HUG. However, in the past decade, no quantitative analyses have been conducted on data from the HUG maternity department to examine access to health care, the quality of health care provided, or obstetrical outcomes among vulnerable migrant pregnant women [30].

Therefore, the main objective of this study was to use existing data to explore potential associations among nationality, legal status, and quality of care with obstetric outcomes. The study began with the hypothesis that legal and migration status are important aspects of vulnerability that affect the quality of care and health outcomes of pregnant women. The underlying objective was to identify characteristics of vulnerable pregnant women in order to inform HUG's ongoing interventions to improve the care provided to this population. A larger study that incorporates three years of data is planned as a follow-up to this exploratory study.

Methodology

Setting and study design

This exploratory cross-sectional retrospective study utilized existing data on all women who delivered at the HUG maternity department in May 2019.

Data collection

Tables 1 and 2 in annex 1 detail the data points that were collected or generated for every woman who delivered at HUG between 1 May and 31 May 2019. Most data points were extracted from HUG's electronic medical records and administrative files in September 2022. Ultrasound records were either drawn from the patient medical records or were manually searched (the latter was mainly the case for undocumented migrant women). The data were anonymized, coded, and stored in a database on the HUG server. The medical records were incomplete for some variables (notably ultrasound, language proficiency, and intimate partner violence). Some variables (such as education level) used unclear or inconsistent classifications. Because the study anonymized the data, we had no access to information that could identify individual participants and could not trace missing data points. Several "missing values" therefore appear in the tables. In addition to the medical record data, public data extracted from

the Swiss Federal Statistical Office (FSO) and OpenStreetMap in January 2023 were used to generate a precarity variable. The ISCO-08[1] structure was used to classify levels of occupation [31].

Defining vulnerability factors

The women included in the study were divided into four categories of legal status: 1) Swiss citizens, 2) documented migrants, 3) undocumented migrants, and 4) asylum seekers. These four groups correspond to three insurance status categories: Swiss citizens and documented migrants with mandatory LaMal insurance, undocumented migrants with no insurance[2], and asylum seekers covered by Swiss Risk & Care.

Precarity status for Swiss and documented migrants was assigned based on the place of residence, combining geographical and neighborhood economic data points[3]. We defined precarity as living in a sub-sector where the median income was below the canton's 2021 minimum wage (of CHF 50,538 per annum) and then designated each patient as either precarious (P) or non-precarious (NP).

Undocumented migrants and asylum seekers were excluded from the geographical analysis due to an assumption of precarity, but also because their addresses are less predictive of their economic status, as residency is mostly attributed or not selected by choice. Women living outside of Geneva and those living in a sub-sector for which income data was not available were not included in the analyses relating to precarious financial situations.

This ultimately resulted in six comparison groups: 1) Swiss non-precarious (SNP) women; 2) Swiss precarious (SP) women; 3) documented migrant non-precarious (DMNP) women; 4) documented migrant precarious (DMP) women; 5) undocumented migrant (UM) women; and 6) women asylum seekers (AS).

[1] The International Standard Classification of Occupation (ISCO-08) determines 8 major groups of occupation with several sub major and minor groups: Managers; Professionals; Technicians and Associate Professionals; Clerical Support Workers; Services and Sales Workers; Skilled Agricultural, Forestry and Fishery Workers; Craft and Related Trades Workers; Stationary Plant and Machine Operators; and Elementary Occupations. This 2007 classification was made by a group of experts on labor statistics [31].

[2] At HUG, undocumented migrants who do not have insurance can access care freely after a social evaluation. They are then labeled administratively as "non billable patients". This label has been used as a surrogate measure for undocumented migrants.

[3] Using each patient's residential address from her medical record, we generated GPS coordinates using the Nominatim search engine from the open-source software OpenStreetMap, available under the Open Database License [32]. The GPS coordinates were then linked with one of the 475 statistical sub-sectors in Geneva canton using data provided by the Territory Information System in Geneva (SITG) [33]. Each patient record was assigned to the median income for a single adult living in their sub-sector using Canton of Geneva official statistics.

Defining independent and dependent variables

Migration status, legal status, and precarity were the independent variables in the analysis (annex 1, table 1). The dependent variables (annex 1, table 2) fell into two categories: quality of care and health outcomes. Two quality-of-care variables were chosen based on international or Swiss recommendations [34]: timely ultrasound (US) (defined as undergoing two ultrasounds, one between 10 and 14 weeks and a second between 18 and 24 weeks of gestation)); and appropriate time for first contact (defined as within the first 12 weeks of pregnancy) [35].

Analysis

All analyses were conducted using the statistical software R (version 4.2.2.). Continuous variables are reported as medians and first and third quartiles, and categorical variables are reported as count and percentages. Comparisons between the documented migrant non-precarious (DMNP) and documented migrant precarious (DMP) women were done using Mann-Whitney U tests for continuous variables, and chi-squared or Fisher's exact tests for categorical variables. A threshold of alpha=0.05 was used to determine statistical significance. The study faced a notable limitation due to the small samples of undocumented migrants (UM) and asylum seekers (AS), which restricted its capacity to achieve statistical significance.

Ethical aspects

The study was approved on 29 August 2022 by the Cantonal Ethics Board of Geneva, Switzerland (N° 2022-01013). The research project was accepted under Article 34 on the re-use of personal health data in the absence of consent.

Results

Study population

As shown in Figure 1, 339 women delivered a newborn (alive or dead, at any gestational age) in the HUG maternity department in May 2019. Swiss patients who were not residents of Geneva canton (n=43) were excluded, leaving 296 patients included in the final analysis. These patients were divided into six groups for the analyses, as shown in Figure 1: Swiss non-precarious (SNP) women (25.3%, n=75), Swiss precarious (SP) women (12.2%, n=36), documented migrant non-precarious (DMNP) women (34.8%, n=103), documented migrant precarious (DMP, n=69) women (23.3%), undocumented migrant (UM) women (2.4%, n=7) and women asylum seekers AS (2.0%, n=6).

Figure 1: Distribution of patient groups

Socio-demographic characteristics of the study population

All demographic data on the study population are included in annex 2, table 3. Figure 2 shows the countries of origin of the women. The majority of both DMNP and DMP women were from Western Europe. UM women came mainly from South America; others migrated from Asia and Africa. AS women migrated mainly from Africa, with some from Asia.

Figure 2: Nationalities of women delivering at the HUG maternity in May 2019

Swiss and documented patients, whether precarious or not, had similar median ages (SNP: 31; SP: 31; DMNP: 32; DMP: 32 years), while the median ages of UMs and ASs were lower (UM: 26; AS: 29 years). Approximately two-thirds of the sample, including more than three-quarters of DMP (75.4%) and AS (83.3%), were delivering their second child. However, among the UMs, more than 50% (4 out of 7) were pregnant for the first time. Parity was highest (2 or more births) in the AS (50.0%), SP (30.6%) and DMP (26.1%).

Regarding marital status, the majority (83.3%) of AS, as well as Swiss and documented migrant women were married (SNP 50.7%, SP 58.3%, DMNP 69.9%, DMP 69.6%). Among the UM women, 57% were single.

French was listed as the first language of more than half of the patients in all groups (including six of the seven UMs). However, a quarter (25.7%) of the patients reportedly did not speak French as either their first or second language.

SNP women had the lowest rate of unemployment (13.3%). Most UM women were not employed (57.1%). The six AS women were also not working, as it is prohibited by Swiss law[4]. Among documented migrants, the fraction of women without occupations was higher in the DMP group (46.4%) compared to the DMNP group (28.2%). The association was opposite among women with high skilled work (35.9% of DMNPs, 20.3% of DMPs, p-value = 0.015) (see annex 3).

[4] In Switzerland, asylum seekers have a “permit N” that does not allow them to work. Documented migrants, including refugees (“permit B”), are allowed to apply for work permits [36].

All Swiss patients (SNP and SP) were insured by LaMAL, as were the majority (over 90%) of both groups of documented migrants (92.2% of DMNP, 98.6% of DMP). A very small proportion of our sample had international insurance (2.9% of DMNP, 1.4% of DMP) or paid out-of-pocket (4.8% of the DMNP). By definition, the six AS women were covered by the Swiss Risk & Care insurance broker; the seven UMs had no insurance and were considered non-billable patients.

Obstetrical outcomes

Complete details on the obstetrical outcomes for the HUG patient population in May 2019 are presented in annex 2, table 4. Outcomes of particular interest include:

Duration of pregnancy

The overall preterm delivery rate was 6.1%. SPs had the highest rate (11.1% of deliveries) of preterm births, followed by SNPs (6.7%). The migrant groups had lower rates of preterm deliveries (DMNPs: 4.8%; DMPs: 5.7%; all pregnancies of UMs and ASs were all full-term).

Pregnancy complications

Despite what might have been predicted based on the literature, in our sample the ASs had no diagnosed complications (such as gestational diabetes/diabetes, preeclampsia, or gestational hypertension) [37-38]. However, it was noted that 66.7% of asylum seekers had undergone female genital cutting (compared to 4% of DMNPs, 6.2% of DMPs and none of the UMs, SPs and SNPs). This condition can lead to complications during delivery, including increased instrumented labor, C-section or postpartum hemorrhage, among others [39].

Type of delivery

All modes of delivery were represented in the six groups. However, vaginal birth was least frequent among Swiss patients regardless of precarity status. None of the AS had C-sections (see Figure 3).

Figure 3: Percentage of type of delivery in each group

Labor complications

Figure 4 shows the prevalence of three maternal complications of labor (type II or III perineal tear, episiotomy, and postpartum hemorrhage) that have strong associations with health outcomes [40]. Episiotomies occurred in

7.8% of cases, with the highest proportion among ASs (33.3%) and none among UM women. Postpartum hemorrhages occurred in 16.2% of women in the sample. In this instance, the UMs had the highest rate (28.6%; 2 out of 7), while no postpartum hemorrhage was recorded among ASs.

Figure 4: Labor complications' variables

Quality of care

The complete table of quality results are detailed in annex 2, table 5. Notable findings include:

First contact with health system

The majority of women in the sample had their first pregnancy-related contacts with the health care system through planned consultations (66.7% for the SNPs and SPs, 73.8% of DMNPs, 72.5% of DMPs, 100% of UMs, 83.3% of ASs). The remaining women first accessed health services during their pregnancies via emergency care (see Figure 5).

Figure 5: Entry point of care at the HUG for patients in each group

Site of routine antenatal care

A majority of Swiss patients (80% of the SNPs and 91.7% of the SPs) attended private antenatal clinics. This proportion was lower for both groups of documented women (DMNPs: 73.8%, DMPs: 66.7%). Among the women with precarious economic status, significantly fewer DMP women used private gynecological monitoring compared with SP women (SP 91.7%, DMP 66.7%, p-value = 0.010). Private antenatal care was used by only one UM woman and none of the AS women.

Timely ultrasound (US) and first contact

Data on "Appropriate time for first contact" and "Timely US" [35] were available for a subset of the sample, namely women who exclusively used HUG's maternity services (i.e., did not use private gynecological clinics) (N=81; SNP n=15, 18.5%; SP n=3, 3.7%; DMNP n=27, 33.3%). Four UM women (66.7%) had an appropriate time for first contact; the other two had their first monitoring visits late, after the first trimester (see table 6). One of these two had neither a timely ultrasound nor an appropriate time for first contact. Over 80% of Swiss patients had a timely ultrasound. On the other hand, we found lower rates of timely ultrasound among both AS (66.7%) and UM women (33.3%).

Discussion

This exploratory study examined the association of three dimensions of vulnerability (either documented or which could be extrapolated from data routinely collected in HUG's electronic medical record system) with key aspects of quality of obstetrical care and obstetrical health outcomes. Some of the results were anticipated while others were more surprising.

Importance of economic status to health and health care

This study was originally conducted to examine associations between a woman's legal and migrant status and obstetrical care quality and outcomes. Our preliminary hypothesis was that women who were undocumented or seeking asylum would have worse obstetrical outcomes than Swiss citizens and documented migrants related, at least in part, to lower quality of care. However, the study revealed that economic precarity, rather than legal status, had the strongest association with several indicators of poor health outcomes.

A surprisingly high proportion (35.5%) of HUG's Swiss or documented obstetric patients face economic precarity (including 12.2% of SP and 23.3% of DMP women). This is almost four times higher than in Switzerland overall in 2019, when 8.7% of the population was described as poor, and 12.2% as having difficulties providing for their needs [41]. This key finding highlights that a patient's economic status may influence their experience with the Swiss healthcare system and health outcomes [42-43]. The study also found associations between a patient's legal status and other factors related to economic precarity, including employment status, marital status, and nation of origin.

Meeting the needs of migrant women during pregnancy and delivery

The study's findings related to both obstetrical outcomes and quality-of-care indicators suggest that HUG is generally meeting the obstetrical care needs of migrant patients. For example, among the obstetrical outcomes examined, we found a lower-than-expected prevalence of labor complications, compared with the latest statistics [44] and no definitive correlation with legal status. Among the patient groups at HUG, those with the highest rates of tearing were UMs (type II: 42.9%, type III: 0.0%), SNPs (type II: 32.0%, type III: 2.7%), and ASs (type II: 33.0%, type III: 0.0%). While lower than expected, these results still suggest that the most vulnerable women are experiencing worse outcomes.

One quality-of-care indicator used in the study is whether a pregnant woman's first contact with the healthcare system during pregnancy is via antenatal care or emergency services. (First healthcare contact during pregnancy via the emergency room can indicate that a woman has no other entry points to health care.) In the study sample, none of the UM women and only one AS woman had first contact via emergency services. The Swiss patients were more likely to use the emergency room for a first contact. This may be partly related to having access to private medical care, in which case women only enter the HUG system for emergency reasons.

The study results suggest that the CAMSCO program seems to be effective in linking undocumented pregnant migrant women to maternity health care in Geneva. This is encouraging, as access to primary care is a positive determinant of follow-up for migrant women in high-income countries [45], and lack of continuity of care has negative effects [46]. However, CAMSCO reaches only approximately 100 women annually. This study's findings on the nature of vulnerability among pregnant women suggest that other programming is required to address the needs of pregnant women facing economic precarity, regardless of their immigration status.

Limitations

The main limitation of this exploratory study was the sample size, which was not powered to generate statistically significant findings when comparing the sub-groups. While these findings can be used as a baseline for comparison for future studies, it is difficult to use them to justify recommendations for intervention. The findings also raise concerns about the quality and consistency of the data collected in HUG's electronic medical records. If the data are incomplete—or cannot be recorded with appropriate nuance—in the current system, it undermines the usefulness of the system and analyses of the data. For example, while the data indicated that French language was commonly spoken in all patient groups, empirical experience raises concerns about the validity of this finding. Inaccurate data on language skills can result in insufficient for translation services during health care [47]. Another questionable finding was that no intimate partner violence was documented. This finding does not align with previous literature [48] or empirical experience. There are several possible explanations for the discrepancy: the data collection process used when completing the electronic patient file may not be conducive to asking sensitive questions, or healthcare workers may decide not to include the data in the file for privacy or security reasons. Yet screening for intimate partner violence during pregnancy is essential, as it is correlated to poor maternal health outcomes [49].

Conclusion

This exploratory study started with the hypothesis that legal status is an important aspect of vulnerability that affects both the quality of care provided to pregnant women and their health outcomes. It explored associations among pregnant women's legal, migration, and precarity statuses, as well as other demographic characteristics, and assessed these characteristics' associations with indicators of health care quality and health outcomes.

Other studies have demonstrated stronger links between migration status and clinical outcomes [50]. This study, however, highlighted that when programs exist to address vulnerability related to women's legal and migration status, economic precarity is a major source of vulnerability. Our findings suggest that HUG is providing quality care to legally vulnerable women, including undocumented women and asylum seekers. This is partly thanks to the CAMSCO program, which coordinates interdisciplinary health interventions for key vulnerable populations in Geneva; it has set precedents for how to integrate socio-economic and cultural support to address social determinants of health [51].

The study also explored other dimensions of vulnerability among the obstetrical patient population at HUG. This led to another key finding: economic precarity, in addition or regardless of legal status, was highly associated with poorer health outcomes and lower quality care for pregnant women.

While these key findings are informative, this was designed as an exploratory study and lacked statistical power. It has provided several important topics for additional research efforts with larger sample sizes and qualitative components. These include further exploration of the relationships among different dimensions of vulnerability, the types of targeted services pregnant women require, and the determinants of health outcomes. Future quantitative studies at HUG should include larger sample sizes to better understand the nature of the associations among demographic characteristics (such as precarity, employment status, language proficiency, and access to private prenatal care) with clinical and quality outcomes. Qualitative research on communication between HUG's migrant patient population and its health care providers could suggest additional ways to improve the quality of care provided to vulnerable patients.

These additional areas of research can further inform improved practices in obstetric care and support at HUG and other hospitals. Potential interventions may include: identifying key variables that should be added to patients' electronic medical records to improve quality of care; additional training for obstetrical staff to improve their understanding and responses to different dimensions of vulnerability; increasing inter-departmental and inter-disciplinary collaboration to more effectively reach and support vulnerable women and their children across the life cycle; and utilization of new technologies, such as artificial intelligence, to mine electronic medical records for more nuanced analyses of both vulnerability and successful approaches to providing quality obstetrical care.

Ethical Review

The study was approved by the Cantonal Ethics Board of Geneva, Switzerland (N° 2022-01013).

Competing Interests

The authors have no competing interests to declare.

References

1. McAuliffe M, Triandafyllidou A. World migration report 2022. Geneva: International Organization for Migration (IOM). 2021.

2. United Nations Department of Economic and Social Affairs PD. International Migration 2020 Highlights. (ST/ESA/SER.A/452). 2020.

3. Office fédéral de la statistique. Population issue de la migration en 2020. 2021. https://www.bfs.admin.ch/news/fr/2021-0186

4. Office cantonal de la statistique (OCSTAT). Migration et Intégration. 2023. https://statistique.ge.ch/domaines/apercu.asp?dom=01_02

5. Office cantonal de la statistique (OCSTAT). Actualités - En 2020, peu de nouvelles demandes d'asile à Genève en raison de la pandémie de Covid-19. 2021. https://statistique.ge.ch/actualites/welcome.asp?actu=4239&mm1=01/01&aaaa1=2021&mm2=02/28&aaaa2=2021&num=0

6. Conseil fédéral. Pour un examen global de la problématique des sans-papiers - Rapport du Conseil fédéral en réponse au postulat de la Commission des institutions politiques du Conseil national du 12 avril 2018 (18.3381). Berne: Confédération Suisse; Dec 2020.

7. Bodenmann, P, Wolff H, Madrid C.Vulnérabilités et santé : pourquoi une nouvelle rubrique?. Rev Med Suisse. 2009/199 (Vol.-5), p. 849–849. https://www.revmed.ch/revue-medicale-suisse/2009/revue-medicale-suisse-

199/vulnerabilites-et-sante-pourquoi-une-nouvelle-rubrique

8. Bodenmann P, Wolff H, Madrid C. Vulnérabilités, diversités et équité en santé. RMS éditions / Médecine et Hygiène. 22 March 2022.

9. Le casse-tête des assurances maladie pour les réfugié·es. Hospice général. 2020. https://www.hospicegeneral.ch/fr/le-casse-tete-des-assurances-maladie-pour-les-refugiees

10. Jackson Y. Health care for undocumented migrants in. Geneva: safety is the key for success. 2018.

11. Improving the health care of pregnant refugee and migrant women and newborn children. Copenhagen: WHO Regional Office for Europe 2018 (Technical guidance on refugee and migrant health). 2018. https://apps.who.int/iris/bitstream/handle/10665/342289/9789289053815-eng.pdf?sequence=1&isAllowed=y

12. The Lancet Public Health. Protecting migrant women. Lancet Public Health. 2018.

13. Philibert M, Deneux-Tharaux C, Bouvier-Colle MH. Can excess maternal mortality among women of foreign nationality be explained by suboptimal obstetric care? BJOG Int J Obstet Gynaecol. Oct 2008.

14. Jackson Y, Courvoisier DS, Duvoisin A, Ferro-Luzzi G, Bodenmann P, Chauvin P, et al. Impact of legal status change on undocumented migrants' health and well-being (Parchemins): protocol of a 4-year, prospective, mixed-methods study. BMJ Open. May 2019.

15. van den Akker T, van Roosmalen J. Maternal mortality and severe morbidity in a migration perspective. Best Pract Res Clin Obstet Gynaecol. Avr 2016.

16. Pedersen GS, Grøntved A, Mortensen LH, Andersen AMN, Rich-Edwards J. Maternal Mortality Among Migrants in Western Europe: A Meta-Analysis. Matern Child Health J. Sept 2014.

17. Fellmeth G, Fazel M, Plugge E. Migration and perinatal mental health in women from low- and middle-income countries: a systematic review and meta-analysis. BJOG Int J Obstet Gynaecol. Avr 2017.

18. Stevenson K, Fellmeth G, Edwards S, Calvert C, Bennett P, Campbell OMR, et al. The global burden of perinatal common mental health disorders and substance use among migrant women: a systematic review and meta-analysis. Lancet Public Health. March 2023.

19. Irwin A, Valentine N, Brown C, et al. The Commission on Social Determinants of Health: Tackling the Social Roots of Health Inequities. PLoS Med. 2006.

20. D'haenens F, Helsloot K, Lauwaert K, et al. Towards an integrated perinatal care pathway for vulnerable women: The development and validation of quality indicators. Midwifery. 2020.

21. Kusuma YS, Kumari R, Kaushal S. Migration and access to maternal healthcare: determinants of adequate

antenatal care and institutional delivery among socio-economically disadvantaged migrants in Delhi, India. Trop Med Int Health. 2013.

22. Schmidt NC, Fargnoli V, Epiney M, Irion O. Barriers to reproductive health care for migrant women in Geneva: a qualitative study. Reprod Health. 2018.

23. Sami J, Quack Lötscher KC, Eperon I, Gonik L, Martinez de Tejada B, Epiney M, et al. Giving birth in Switzerland: a qualitative study exploring migrant women's experiences during pregnancy and childbirth in Geneva and Zurich using focus groups. Reprod Health. Dec 2019.

24. Bollini P, Wanner P, Pampallona S. Trends in maternal mortality in Switzerland among Swiss and foreign nationals, 1969-2006. Int J Public Health. 2011.

25. République et Canton de Genève. ge.ch. 2019. Opération Papyrus. https://www.ge.ch/taxonomy/term/564

26. Huschke S. Fragile Fabric: Illegality Knowledge, Social Capital and Health-seeking of Undocumented Latin American Migrants in Berlin. J Ethn Migr Stud. 2014.

27. Keygnaert I, Ivanova O, Guieu A, et al. What is the evidence on the reduction of inequalities in accessibility and quality of maternal health care delivery for migrants? - A review of the existing evidence in the WHO European Region. WHO Health Evidence Network synthesis report 45. 2016.

28. Hôpitaux Universitaires de Genève (HUG). Activité médicale – Chiffres clés 2019. 4 May 2021. https://www.hug.ch/chiffres-cles-2019/activite-medicale

29. Office cantonal de la statistique (OCSTAT). Bilan et état de la population du canton de Genève en 2019. 5 mars 2020.

30. Wolff H, Epiney M, Lourenco AP, et al. Undocumented migrants lack access to pregnancy care and prevention. BMC Public Health. 2008.

31. ISCO - International Standard Classification of Occupations. https://www.ilo.org/public/english/bureau/stat/isco/isco08/

32. OpenStreetMap. https://www.openstreetmap.org/copyright

33. SITG - The territorial information system of Geneva | opendata.swiss. https://opendata.swiss/en/organization/sitg-systeme-dinformation-du-territoire-a-geneve#:~:text=The%20territorial%20information%20system%20of%20Geneva%20(SITG)%20is%20an%20organization,of%20the%20territory%20of%20Geneva

34. Hôpitaux Universitaires de Genève (HUG). Mon calendrier de grossesse. https://www.hug.ch/sites/interhug/files/structures/obstétrique/documents/calendrier_grossesse_7.pdf

34. World Health Organization. WHO recommendations on antenatal care for a positive pregnancy experience. Geneva: World Health Organization. 2016. 152 p. https://apps.who.int/iris/handle/10665/250796

36. Organisation Suisse d'Aide aux Réfugiés. Statut de séjour. 2023. https://www.osar.ch/themes/asile-en-suisse/statut-de-sejour

37. Ajjarapu A, Story WT, Haugsdal M. Addressing Obstetric Health Disparities among Refugee Populations: Training the Next Generation of Culturally Humble OB/GYN Medical Providers. Teach Learn Med. 27 May 2021.

38. Hayes I, Enohumah K, McCaul C. Care of the migrant obstetric population. Int J Obstet Anesth. Oct 2011.

39. World Health Organization. Female genital mutilation. 2023. https://www.who.int/news-room/fact-sheets/detail/female-genital-mutilation

40. Stranges E. Complicating Conditions of Vaginal Deliveries and Cesarean Sections. 2009.

41. Office fédéral de la statistique OFS. La pauvreté en Suisse a continué de progresser en 2019 - Pauvreté, distribution des revenus et conditions de vie en 2019. 18 Feb 2021. https://www.bfs.admin.ch/asset/fr/15344809

42. Feinstein JS. The Relationship between Socioeconomic Status and Health: A Review of the Literature. Milbank Q. 1993.

43. Meraya AM, Dwibedi N, Tan X, Innes K, Mitra S, Sambamoorthi U. The dynamic relationships between economic status and health measures among working-age adults in the United States. Health Econ. Aug 2018.

44. Office fédéral de la statistique OFS. Recul du recours aux césariennes et aux épisiotomies en 2017 - Accouchements et santé maternelle en 2017. 17 May 2019. https://www.bfs.admin.ch/asset/fr/8288948

45. Owens C, Dandy J, Hancock P. Perceptions of pregnancy experiences when using a community-based antenatal service: A qualitative study of refugee and migrant women in Perth, Western Australia. Women Birth. Avr 2016.

46. Sami J, Quack Lötscher KC, Eperon I, Gonik L, Martinez de Tejada B, Epiney M, et al. Giving birth in Switzerland: a qualitative study exploring migrant women's experiences during pregnancy and childbirth in Geneva and Zurich using focus groups. Reprod Health. Dec 2019.

47. Rizzo N, Ciardelli V, Gandolfi Colleoni G, Bonavita B, Parisio C, Farina A, et al. Delivery and immigration: the experience of an Italian hospital. Eur J Obstet Gynecol Reprod Biol. Oct 2004.

48. Sobotka T. Overview Chapter 7: The rising importance of migrants for childbearing in Europe. Demogr Res. 1 July 2008.

49. Bianchi AL, Cesario SK, McFarlane J. Interrupting Intimate Partner Violence During Pregnancy With an

Effective Screening and Assessment Program. J Obstet Gynecol Neonatal Nurs. July 2016.

50. Alder J, Fink N, Lapaire O, Urech C, Meyer A, Bitzer J, et al. The effect of migration background on obstetric performance in Switzerland. Eur J Contracept Reprod Health Care. Jan 2008.

51. Hôpitaux Universitaires de Genève (HUG). Mission et prestations - Consultation ambulatoire mobile de soins communautaires (CAMSCO). https://www.hug.ch/medecine-premier-recours/mission-prestations-camsco

Annexes

Annex 1: Description of included variables

Table 1: Independent variables

Variable	Options	Source data
Migration status	Migrant (M)<br>Swiss (S):	Nationality variable: Switzerland = S, all others = M
Legal status	Documented (D)<br>Undocumented (U)	????
*Precarity**	Precarious (P)<br>Non-precarious (NP)	All UMs and ASs were characterized as precarious.<br><br>For Swiss and documented migrants, precarity was calculated as follows: Patients' residential addresses were used to generate GPS coordinates (using the Nominatim search engine from the open-source software OpenStreetMap, available under the Open Database License) [32]. Using data provided by the Territory Information System in Geneva (SITG) (accessed on February 1, 2023) [33], the GPS coordinates were linked with one of the 475 sub-sectors in Geneva canton and assigned the median income for a single adult living in that sub-sector. Precarity was then defined as below the defined cantonal minimum wage.

Table 2: Demographic and dependent variables

Category	Variable	Options (continuous or discrete)	Reason for inclusion
**Socio-demographic variables**	*Age of patient*	Continuous	Known association with health outcomes & data available from records
	Parity	Continuous	Known association with health outcomes & data available from records
	Gravidity	Continuous	Known association with health outcomes & data available from records
	Nationality (Swiss or by continent)	Discrete	Data available from records
	Tariff attribute	Discrete	Data available from records
	French language acquisition	Discrete	Data available from records
	Marital status	Discrete	Data available from records
	Religion	Discrete	Data available from records
	Profession	Discrete	Data available from records
	Active smoker	Discrete	Known association with health outcomes & data available from records
**Obstetrical variables**	*Term*	Discrete	Known association with health outcomes & data available from records
	Delivery type	Discrete	Known association with health outcomes & data available from records
	Induced labor	Discrete	Known association with health outcomes & data available from records

Category	Variable	Options (continuous or discrete)	Reason for inclusion
	Past C-section	Discrete	Known association with health outcomes & data available from records
	Single or multiple pregnancy	Discrete	Known association with health outcomes & data available from records
	Anesthesia	Discrete	Data available from records
	Postpartum hemorrhage	Discrete	Known association with health outcomes & data available from records
	Perineal tear	Discrete	Known association with health outcomes & data available from records
	Episiotomy	Discrete	Known association with health outcomes & data available from records
	Threat of preterm delivery	Discrete	Known association with health outcomes & data available from records
	Gestational diabetes or diabetes	Discrete	Known association with health outcomes & data available from records
	Gestational hypertension	Discrete	Data available from records
	Eclampsia or preeclampsia	Discrete	Known association with health outcomes & data available from records
	Female genital cutting	Discrete	Known association with health outcomes & data available from records
	Newborn's birth weight	Continuous	Known association with health outcomes & data available from records

Category	Variable	Options (continuous or discrete)	Reason for inclusion
	Newborn’s hospitalization in the neonatal unit - main diagnosis	Discrete	Known association with health outcomes & data available from records
**Quality variables**	*First contact: with hospital emergency room*	Discrete	Data available from records
	Private gynecological monitoring	Discrete	Data available from records
	Folic acid supplementation	Discrete	Known association with health outcomes & data available from records
	Breastfeeding	Discrete	Known association with health outcomes & data available from records
	Admission motive	Discrete	Data available from records
	Timely ultrasound	Discrete	Data available from records
	Appropriate time for the first contact for the UMs	Discrete	Data available from records

Annex 2: Tables of results, by group

Table 3: Sociodemographic variables, by group

**Socio-demographic variable**	**Total (n= 296)**	**Swiss non-precarious SNP (n= 75, 25.3%)**	**Swiss precarious SP (n=36, 12.2%)**	**Documented migrant non-precarious DMNP (n=103, 34.8%)**	**Documented migrant precarious DMP (n=69, 23.3%)**	**Undocumented migrants UM (n=7, 2.4%)**	**Asylum seekers AS (n=6, 2.0%)**
Age of patient							
	31 (28 - 36)	31 (28 - 35)	31 (29 - 34.2)	32 (29 - 36)	32 (27 - 37)	26 (25 - 33.5)	29 (27.5 - 30.5)
Parity							
0	134 (45.3%)	42 (53.3%)	16 (44.4%)	47 (45.6%)	26 (37.7%)	4 (57.1%)	1 (16.7%)
1	101 (34.1%)	26 (34.7%)	8 (22.2%)	38 (36.9%)	25 (36.2%)	2 (28.6%)	2 (33.3%)
2+	61 (20.6%)	9 (12.0%)	12 (33.3%)	18 (17.5%)	18 (26.1%)	1 (14.3%)	3 (50.0%)
Gravidity							
1	99 (33.4%)	32 (42.7%)	11 (30.6%)	34 (32.0%)	17 (24.6%)	4 (57.1%)	1 (16.7%)
2+	197 (66.6%)	43 (57.3%)	25 (69.4%)	69 (67.0%)	52 (75.4%)	3 (42.9%)	5 (83.3%)
Nationality (Swiss or by continent)							
Swiss	111 (37.5%)	75 (100%)	36 (100%)	0	0	0	0
Africa	35 (11.8%)	0	0	14 (13.6%)	16 (23.2%)	1 (14.3%)	4 (66.7%)
North America	3 (1.0%)	0	0	2 (1.9%)	1 (1.4%)	0	0
South America	26 (8.8%)	0	0	15 (14.6%)	7 (10.1%)	4 (57.1%)	0
Asia	19 (6.4%)	0	0	9 (8.7%)	6 (8.7%)	2 (28.6%)	2 (33.3%)
Eastern Europe	25 (8.4%)	0	0	13 (12.6%)	12 (17.4%)	0	0
Western Europe	77 (26.0%)	0	0	50 (48.5%)	27 (39.1%)	0	0
French language acquisition							
1st	179 (60.5%)	45 (60.0%)	23 (63.9%)	60 (58.3%)	41 (59.4%)	6 (85.7%)	4 (66.7%)
2nd	41 (13.9%)	13 (17.3%)	4 (11.1%)	15 (14.6%)	9 (13.0%)	0	0
No mention either as a 1st or 2nd language	76 (25.7%)	17 (22.7%)	9 (25.0%)	28 (27.2%)	19 (27.5%)	1 (14.3%)	2 (33.3%)
Tariff attribute							
no data	17 (5.7%)	5 (6.7%)	3 (8.3%)	5 (4.9%)	4 (5.8%)	0	0
Confederate domiciled in	93 (31.4%)	64 (85.3%)	29 (80.6%)	0	0	0	0

Socio-demographic variable	Total (n= 296)	Swiss non-precarious SNP (n= 75, 25.3%)	Swiss precarious SP (n=36, 12.2%)	Documented migrant non-precarious DMNP (n=103, 34.8%)	Documented migrant precarious DMP (n=69, 23.3%)	Undocumented migrants UM (n=7, 2.4%)	Asylum seekers AS (n=6, 2.0%)
the canton							
Foreigner domiciled in the canton	175 (59.1%)	6 (8.0%)	4 (11.1%)	90 (87.4%)	63 (91.3%)	6 (85.7%)	6 (100%)
Foreigner domiciled abroad	1 (0.3%)	0	0	0	1 (1.4%)	0	0
Borderer	1 (0.3%)	0	0	1 (1.0%)	0	0	0
International officer/mission/consulate	8 (2.7%)	0	0	7 (6.8%)	1 (1.4%)	0	0
Domiciled in Geneva, no LAMal	1 (0.3%)	0	0	0	0	1 (14.3%)	0
Marital status							
Single	92 (31.1%)	33 (44.0%)	12 (33.3%)	25 (24.3%)	17 (24.6%)	4 (57.1%)	1 (16.7%)
In a relationship (concubinage)	4 (1.4%)	1 (1.3%)	0	1 (1.0%)	2 (2.9%)	0	0
Married	186 (62.8%)	38 (50.7%)	21 (58.3%)	72 (69.9%)	48 (69.6%)	2 (28.6%)	5 (83.3%)
Separated/divorced	13 (4.4%)	3 (4.0%)	3 (8.3%)	4 (3.9%)	2 (2.9%)	1 (14.3%)	0
Widow	1 (0.3%)	0	0	1 (1.0%)	0	0	0
Religion							
No religion	70 (23.6%)	24 (32.0%)	11 (30.6%)	22 (21.4%)	13 (18.8%)	0	0
Buddhism	3 (1.0%)	0	1 (2.8%)	0	1 (1.4%)	1 (14.3%)	0
Christian	106 (35.8%)	35 (46.7%)	11 (30.6%)	39 (37.9%)	19 (27.5%)	2 (28.6%)	0
Hinduism	2 (0.7%)	0	0	0	2 (2.9%)	0	0
Judaism	1 (0.3%)	0	0	1 (1.0%)	0	0	0
Muslim	44 (14.9%)	5 (6.7%)	6 (16.7%)	15 (14.6%)	15 (21.7%)	1 (14.3%)	2 (33.3%)
Other	5 (1.7%)	2 (2.7%)	1 (2.8%)	1 (1.0%)	1 (1.4%)	0	0
Refusal to answer	10 (3.4%)	2 (2.7%)	2 (5.6%)	5 (4.9%)	1 (1.4%)	0	0
Not asked	55 (18.6%)	7 (9.3%)	4 (11.1%)	20 (19.4%)	17 (24.6%)	3 (42.9%)	4 (66.7%)
Profession							
No work	91 (30.7%)	10 (13.3%)	10 (27.8%)	29 (28.2%)	32 (46.4%)	4 (57.1%)	6 (100%)
Student	13 (4.4%)	4 (5.3%)	2 (5.6%)	6 (5.8%)	0	1 (14.3%)	0

Socio-demographic variable	Total (n= 296)	Swiss non-precarious SNP (n= 75, 25.3%)	Swiss precarious SP (n=36, 12.2%)	Documented migrant non-precarious DMNP (n=103, 34.8%)	Documented migrant precarious DMP (n=69, 23.3%)	Undocumented migrants UM (n=7, 2.4%)	Asylum seekers AS (n=6, 2.0%)
Domestic work	13 (4.4%)	0	0	7 (6.8%)	6 (8.7%)	0	0
Low skill work	81 (27.4%)	27 (36.0%)	12 (33.3%)	24 (23.3%)	17 (24.6%)	1 (14.3%)	0
High skill work	97 (32.8%)	34 (45.3%)	12 (33.3%)	37 (35.9%)	14 (20.3%)	0	0
No data	1 (0.3%)	0	0	0	0	1 (14.3%)	0
Active smoker							
Yes	20 (6.8%)	6 (8.0%)	4 (11.1%)	3 (2.9%)	7 (10.6%)	0	0
No	273 (93.2%	69 (92.0%)	32 (88.9%)	100 (97.1%)	59 (89.4%)	7 (100%)	6 (100%)
missing values	3				3		

Table 4: Obstetrical variables, by group

Obstetrical variables	Total (n= 296)	Swiss non precarious SNP (n=75, 25.3%)	Swiss precarious SP (n=36, 12.2%)	Documented migrant non precarious DMNP (n=103, 34.8%)	Documented migrant precarious DMP (n=69, 23.3%)	Undocumented migrants UM (n=7, 2.4%)	Asylum seekers AS (n=6, 2.0%)
Term							
Preterm (<37 SA)	18 (6.1%)	5 (6.7%)	4 (11.1%)	5 (4.8%)	4 (5.7%)	0	0
Term (37-41 6/7 SA)	278 (93.9%)	70 (93.3%)	32 (88.9%)	98 (95.1%)	65 (94.2%)	7 (100%)	6 (100%)
Delivery type							
Spontaneous vaginal birth	178 (60.1%)	38 (50.7%)	21 (58.3%)	62 (60.2%)	47 (68.1%)	5 (71.4%)	5 (83.3%)
Instrumented vaginal birth	41 (13.9%)	17 (22.7%)	5 (13.9%)	9 (8.7%)	9 (13.0%)	0	1 (16.7%)
Cesarean section (C-section)	77 (26.0%)	20 (26.7%)	10 (27.8%)	32 (31.1%)	13 (18.8%)	2 (28.6%)	0
Induced labor							
No	123 (41.6%)	30 (40.0%)	14 (38.9%)	44 (42.7%)	28 (40.6%)	4 (57.1%)	3 (50.0%)
Yes	132 (44.6%)	34 (45.3%)	16 (44.4%)	44 (42.7%)	33 (47.8%)	2 (28.6%)	3 (50.0%)
Cesarean section before labor	41 (13.9%)	11 (14.7%)	6 (16.7%)	15 (14.6%)	8 (11.6%)	1 (14.3%)	0
Past C-section							
Yes	36 (12.2%)	8 (10.7%)	8 (22.2%)	11 (10.7%)	7 (10.1%)	1 (14.3%)	1 (16.7%)
No	260 (87.8%)	67 (89.3%)	28 (77.8%)	92 (89.3%)	62 (89.9%)	6 (85.7%)	5 (83.3%)
Single or multiple pregnancy							
Multiple	5 (1.7%)	3 (4.0%)	1 (2.8%)	0	1 (1.4%)	0	0
Single	291 (98.3%)	72 (96.0%)	35 (97.2%)	103 (100%)	68 (98.6%)	7 (100%)	6 (100%)
Anesthesia							
No	35 (11.8%)	6 (8.0%)	5 (13.9%)	19 (18.4%)	4 (5.8%)	0	1 (16.7%)
Gas (EMONO	8 (2.7%)	4 (5.3%)	0	1 (1.0%)	3 (4.3%)	0	0
Epidural	206 (69.6%)	56 (74.7%)	24 (66.7%)	70 (68.0%)	51 (73.9%)	4 (57.1%)	1 (16.7%)
General	7 (2.4%)	0	0	3 (2.9%)	4 (5.8%)	0	0
Other/not described	40 (13.5%)	9 (12.0%)	7 (19.4%)	10 (9.7%)	7 (10.1%)	3 (42.9%)	4 (66.7%)

Obstetrical variables	Total (n= 296)	Swiss non precarious SNP (n=75, 25.3%)	Swiss precarious SP (n=36, 12.2%)	Documented migrant non precarious DMNP (n=103, 34.8%)	Documented migrant precarious DMP (n=69, 23.3%)	Undocumented migrants UM (n=7, 2.4%)	Asylum seekers AS (n=6, 2.0%)
Postpartum hemorrhage							
Yes	48 (16.2%)	10 (13.3%)	6 (16.7%)	19 (18.4%)	11 (15.9%)	2 (28.6%)	0
No	248 (83.8%)	65 (86.7%)	30 (83.3%)	84 (81.6%)	58 (84.1%)	5 (71.4%)	6 (100%)
Perineal tear							
None	163 (55.1%)	37 (49.3%)	22 (61.1%)	60 (58.3%)	37 (53.6%)	4 (57.1%)	3 (50.0%)
Type I	61 (20.6%)	12 (16.0%)	6 (16.7%)	25 (24.3%)	17 (24.6%)	0	1 (16.7%)
Type II	69 (23.3%)	24 (32.0%)	8 (22.2%)	17 (16.5%)	15 (21.7%)	3 (42.9%)	2 (33.3%)
Type III	3 (1.0%)	2 (2.7%)	0	1 (1.0%)	0	0	0
Episiotomy							
Yes	23 (7.8%)	8 (10.7%)	4 (11.1%)	4 (3.9%)	5 (7.2%)	0	2 (33.3%)
No	273 (92.2%)	67 (89.3%)	32 (88.9%)	99 (96.1%)	64 (92.8%)	7 (100%)	4 (66.7%)
Threat of preterm delivery							
Yes	8 (2.8%)	1 (1.4%)	1 (3.0%)	4 (4.1%)	2 (3.1%)	0	0
No	274 (97.2%)	73 (98.6%)	32 (97.0%)	94 (95.9%)	62 (96.9%)	7 (100%)	6 (100%)
missing values	14	1	3	5	5		
Gestational diabetes or diabetes							
Yes	29 (9.8%)	6 (8.0%)	7 (19.4%)	10 (9.7%)	5 (7.2%)	1 (14.3%)	0
No	267 (90.2%)	69 (92.0%)	29 (80.6%)	93 (90.3%)	64 (92.8%)	6 (85.7%)	6 (100%)
Gestational hypertension							
Yes	10 (3.4%)	1 (1.3%)	4 (11.1%)	2 (1.9%)	2 (2.9%)	1 (14.3%)	0
No	286 (96.6%)	74 (98.7%)	32 (88.9%)	101 (98.1%)	67 (97.1%)	6 (85.7%)	6 (100%)
Eclampsia or preeclampsia							
Yes	10 (3.4%)	2 (2.7%)	2 (5.6%)	1 (1.0%)	4 (5.8%)	1 (14.3%)	0
No	286 (96.6%)	73 (97.3%)	34 (94.4%)	102 (99.0%)	65 (94.2%)	6 (85.7%)	6 (100%)
Female genital cutting							

**Obstetrical variables**	**Total (n= 296)**	**Swiss non precarious SNP (n=75, 25.3%)**	**Swiss precarious SP (n=36, 12.2%)**	**Documented migrant non precarious DMNP (n=103, 34.8%)**	**Documented migrant precarious DMP (n=69, 23.3%)**	**Undocumented migrants UM (n=7, 2.4%)**	**Asylum seekers AS (n=6, 2.0%)**
present							
Yes	12 (4.3%)	0	0	4 (4.1%)	4 (6.2%)	0	4 (66.7%)
No	270 (95.7%)	74 (100%)	33 (100%)	94 (95.9%)	60 (93.8%)	7 (100%)	2 (33.3%)
missing values	14	1	3	5	5		
Newborn’s birth weight							
Low (<2500 g)	21 (7.1%)	6 (8.0%)	3 (8.3%)	8 (7.8%)	3 (4.3%)	1 (14.3%)	0
Normal (2500-3500 g)	258 (87.2%)	63 (84.0%)	31 (86.1%)	89 (86.4%)	63 (91.3%)	6 (85.7%)	6 (100%)
High (>4000 g)	17 (5.7%)	6 (8.0%)	2 (5.6%)	6 (5.8%)	3 (4.3%)	0	0
Newborn’s hospitalization in the neonatal unit - Main diagnosis							
None	286 (96.6%)	74 (98.7%)	31 (86.1%)	101 (98.1%)	67 (97.1%)	7 (100%)	6 (100%)
Neonatal respiratory distress syndrome	3 (1.0%)	0	1 (2.8%)	1 (1.0%)	1 (1.4%)	0	0
Transient tachypnea in newborn	1 (0.3%)	0	1 (2.8%)	0	0	0	0
Severe obstetric asphyxia	1 (0.3%)	0	0	1 (1.0%)	0	0	0
Other neonatal respiratory distress	1 (0.3%)	0	1 (2.8%)	0	0	0	0
Low birth weight	1 (0.3%)	0	0	0	1 (1.4%)	0	0
Extreme prematurity	2 (0.7%)	1 (1.3%)	1 (2.8%)	0	0	0	0
Twin pregnancy	1 (0.3%)	0	1 (2.8%)	0	0	0	0

Table 5: Quality variables, by group

Quality variables	Total (n= 296)	Swiss non precarious SNP (n=75, 25.3%)	Swiss precarious SP (n=36, 12.2%)	Documented migrant non precarious DMNP (n=103, 34.8%)	Documented migrant precarious DMP (n=69, 23.3%)	Undocumented migrants UM (n=7, 2.4%)	Asylum seekers AS (n=6, 2.0%)
First contact with hospital: emergency room							
No	212 (71.6%)	50 (66.7%)	24 (66.7%)	76 (73.8%)	50 (72.5%)	7 (100%)	5 (83.3%)
Yes	84 (28.4%)	25 (33.3%)	12 (33.3%)	27 (26.2%)	19 (27.5%)	0	1 (16.7%)
Private gynecological monitoring							
No	80 (27.0%)	15 (20.0%)	3 (8.3%)	27 (26.2%)	23 (33.3%)	6 (85.7%)	6 (100%)
Yes	216 (73.0%)	60 (80.0%)	33 (91.7%)	76 (73.8%)	46 (66.7%)	1 (14.3%)	0
Folic acid supplementation							
No	185 (64.2%)	48 (64.0%)	21 (60.0%)	66 (66.0%)	42 (64.6%)	3 (42.9%)	5 (83.3%)
Yes	103 (35.8%)	27 (36.0%)	14 (40.0%)	34 (34.0%)	23 (35.4%)	4 (57.1%)	1 (16.7%)
missing values	8		1	3	4		
Breastfeeding							
Bottle	49 (16.6%)	13 (17.3%)	4 (11.1%)	18 (17.5%)	14 (20.3%)	0	0
Breastfeeding	247 (83.4%)	62 (82.7%)	32 (88.9%)	85 (82.5%)	55 (79.7%)	7 (100%)	6 (100%)
Admission motive							
Bleeding/3rd trimester hemorrhage	4 (1.4%)	2 (2.7%)	0	1 (1.0%)	1 (1.4%)	0	0
CS	1 (0.3%)	0	0	1 (1.0%)	0	0	0
Decrease in fetal movements	1 (0.3%)	0	0	1 (1.0%)	0	0	0
Oligohydramnios /anamnios	1 (0.3%)	0	0	1 (1.0%)	0	0	0
Other	1 (0.3%)	1 (1.3%)	0	0	0	0	0
Preeclampsia (PE)/suspicion of PE	5 (1.7%)	0	2 (5.6%)	1 (1.0%)	2 (2.9%)	0	0

Quality variables	Total (n= 296)	Swiss non precarious SNP (n=75, 25.3%)	Swiss precarious SP (n=36, 12.2%)	Documented migrant non precarious DMNP (n=103, 34.8%)	Documented migrant precarious DMP (n=69, 23.3%)	Undocumented migrants UM (n=7, 2.4%)	Asylum seekers AS (n=6, 2.0%)
Pre-labor	5 (1.7%)	1 (1.3%)	0	2 (2.0%)	1 (1.4%)	1 (14.3%)	0
Premature or spontaneous rupture of membrane	41 (13.9%)	13 (17.3%)	6 (16.7%)	13 (12.7%)	7 (10.1%)	1 (14.3%)	1 (16.7%)
Spontaneous/maturation/ trigger	228 (77.3%)	56 (74.7%)	26 (72.2%)	78 (76.5%)	58 (84.1%)	5 (71.4%)	5 (83.3%)
Suspicious of pathological CTG	5 (1.7%)	1 (1.3%)	1 (2.8%)	3 (2.9%)	0	0	0
Termination of pregnancy/ death in utero	1 (0.3%)	0	0	1 (1.0%)	0	0	0
Threat of preterm delivery	2 (0.7%)	1 (1.3%)	1 (2.8%)	0	0	0	0
missing values	1			1			

Table 6: Quality variables for women with no private care

Quality variables	Total (n=81)	Swiss non precarious SNP (n=15, 18.5%)	Swiss precarious SP (n=3, 3.7%)	Documented migrant non precarious DMNP (n=27, 33.3%)	Documented migrant precarious DMP (n=24, 29.6%)	Undocumented migrants UM (n=6, 7.4%)	Asylum seekers AS (n=6, 7.4%)
Timely ultrasound							
Yes	59 (73.8%)	13 (86.7%)	3 (100%)	19 (70.4%)	18 (78.3%)	4 (66.7%)	2 (33.3%)
No	21 (26.2%)	2 (13.3%)	0	8 (29.6%)	5 (21.7%)	2 (33.3%)	4 (66.7%)
Appropriate time for first contact							
Yes	4 (66.7%)	0	0	0	0	4 (66.7%)	0
No	2 (33.3%)	0	0	0	0	2 (33.3%)	0
missing values	75	15	3	27	24		6

Annex 3: Tables of results among documented migrant women, by precarity status

Table 7: Socio-demographic variables, precarious vs. non-precarious documented women

Socio-demographic variables	Documented migrant non precarious DMNP (n=103, 59.9%)	Documented migrant precarious DMP (n=69, 40.1%)	p-value (Mann-Whitney/chi^2)
Age of patient	32 (29 - 36)	32 (27 - 37)	0.570
Parity			0.383
0	47 (45.6%)	26 (37.7%)	
1	38 (36.9%)	25 (36.2%)	
2+	18 (17.5%)	18 (26.1%)	
Gravidity			0.313[1]
1	34 (33.0%)	17 (24.6%)	
2+	69 (67.0%)	52 (75.4%)	
Tariff attribute			0.353[1]
no data	5 (4.9%)	4 (5.8%)	
Foreigner domiciled in the canton	1 (1.0%)	0	
Foreigner domiciled abroad	89 (86.4%)	63 (91.3%)	
Borderer	0	1 (1.4%)	
International officer/mission/consulate	1 (1.0%)	0	
Domiciled in Geneva, no LAMal	7 (6.8%)	1 (1.4%)	
French acquisition			0.961
1st	60 (58.3%)	41 (59.4%)	
2nd	15 (14.6%)	9 (13.0%)	
no mention	28 (27.2%)	19 (27.5%)	
Marital status			0.909[1]
0	25 (24.3%)	17 (24.6%)	

Socio-demographic variables	Documented migrant non precarious DMNP (n=103, 59.9%)	Documented migrant precarious DMP (n=69, 40.1%)	p-value (Mann-Whitney/chi²)
1	1 (1.0%)	2 (2.9%)	
2	72 (69.9%)	48 (69.6%)	
3	4 (3.9%)	2 (2.9%)	
4	1 (1.0%)	0	
Religion			0.214[1]
No religion	22 (21.4%)	13 (18.8%)	
Buddhism	0	1 (1.4%)	
Christian	40 (38.8%)	19 (27.5%)	
Hinduism	0	2 (2.9%)	
Judaism	1 (1.0%)	0	
Muslim	15 (14.6%)	15 (21.7%)	
Other	1 (1.0%)	1 (1.4%)	
Refusal to answer	5 (4.9%)	1 (1.4%)	
Not asked	19 (18.4%)	17 (24.6%)	
Profession			0.015[1]
No work	29 (28.2%)	32 (46.4%)	
Student	6 (5.8%)	0	
Domestic work	7 (6.8%)	6 (8.7%)	
Low skill work	23 (22.3%)	17 (24.6%)	
High skill work	38 (36.9%)	14 (20.3%)	
Active smoker			0.049[1]
Yes	100 (97.1%)	59 (89.4%)	

Socio-demographic variables	Documented migrant non precarious DMNP (n=103, 59.9%)	Documented migrant precarious DMP (n=69, 40.1%)	p-value (Mann-Whitney/chi^2)
No	3 (2.9%)	7 (10.6%)	
missing values		3	

[1]Fisher's exact

Table 8: Obstetrical variables comparing precarious and non-precarious declared women

Obstetrical variables	Documented migrant non precarious DMNP (n=103, 59.9%)	Documented migrant precarious DMP (n=69, 40.1%)	p-value (Mann-Whitney/chi^2)
Term			0.863[1]
Preterm (<37 SA)	5 (4.8%)	4 (5.7%)	
Term (37-41 6/7 SA)	98 (95.1%)	65 (94.2%)	
Delivery type			0.192
Spontaneous vaginal birth	61 (59.2%)	47 (68.1%)	
Instrumented vaginal birth	10 (9.7%)	9 (13.0%)	
Cesarean section	32 (31.1%)	13 (18.8%)	
Induced labor			0.700
No	45 (43.7%)	28 (40.6%)	
Yes	43 (41.7%)	33 (47.8%)	
Cesarean section before labor	15 (14.6%)	8 (11.6%)	
Past CS			0.999
No	92 (89.3%)	62 (89.9%)	
Yes	11 (10.7%)	7 (10.1%)	
Single or multiple pregnancy			0.401[1]
No	103 (100%)	68 (98.6%)	
Yes	0	1 (1.4%)	
Anesthesia			0.097[1]
No	18 (17.5%)	4 (5.8%)	
Gaz (EMONO)	1 (1.0%)	3 (4.3%)	
Epidural	71 (68.9%)	51 (73.9%)	
General	3 (2.9%)	4 (5.8%)	

**Obstetrical variables**	**Documented migrant non precarious DMNP (n=103, 59.9%)**	**Documented migrant precarious DMP (n=69, 40.1%)**	**p-value (Mann-Whitney/chi^2)**
Other/not described	10 (9.7%)	7 (10.1%)	
Postpartum hemorrhage			0.826
No	84 (81.6%)	58 (84.1%)	
Yes	19 (18.4%)	11 (15.9%)	
Perineal tear			0.852[1]
None	60 (58.3%)	37 (53.6%)	
Type I	24 (23.3%)	17 (24.6%)	
Type II	18 (17.5%)	15 (21.7%)	
Type III	1 (1.0%)	0	
Episiotomy			0.525[1]
Yes	98 (95.1%)	64 (92.8%)	
No	5 (4.9%)	5 (7.2%)	
Threat of preterm delivery			0.999[1]
No	94 (95.9%)	62 (96.9%)	
Yes	4 (4.1%)	2 (3.1%)	
missing values	5	5	
Gestational diabetes or diabetes			0.775
No	93 (90.3%)	64 (92.8%)	
Yes	10 (9.7%)	5 (7.2%)	
Gestational hypertension			0.999[1]
No	101 (98.1%)	67 (97.1%)	
Yes	2 (1.9%)	2 (2.9%)	

Obstetrical variables	Documented migrant non precarious DMNP (n=103, 59.9%)	Documented migrant precarious DMP (n=69, 40.1%)	p-value (Mann-Whitney/chi^2)
Eclampsia or preeclampsia			0.159[1]
No	102 (99.0%)	65 (94.2%)	
Yes	1 (1.0%)	4 (5.8%)	
Female genital cutting			0.713[1]
No	94 (95.9%)	60 (93.8%)	
Yes	4 (4.1%)	4 (6.2%)	
missing values	5	5	
Newborn's birth weight			0.711[1]
Low (<2500 g)	8 (7.8%)	3 (4.3%)	
Normal (2500-3500 g)	89 (86.4%)	63 (91.3%)	
High (4000 g)	6 (5.8%)	3 (4.3%)	
Newborn's hospitalization in the neonatal unit - Main diagnosis			0.701[1]
none	101 (98.1%)	67 (97.1%)	
neonatal respiratory distress syndrome	1 (1.0%)	1 (1.4%)	
severe obstetric asphyxia	1 (1.0%)	0	
low birth weight	0	1 (1.4%)	

[1]Fisher's exact

Table 9: Quality variables, precarious vs. non-precarious documented women

Quality variables	Documented migrant non precarious (n=103, 59.9%)	Documented migrant precarious (n=69, 40.1%)	p-value (Mann-Whitney/chi^2)
First contact with hospital: emergency room			0.874

Quality variables	Documented migrant non precarious (n=103, 59.9%)	Documented migrant precarious (n=69, 40.1%)	p-value (Mann-Whitney/chi^2)
No	77 (74.8%)	50 (72.5%)	
Yes	26 (25.2%)	19 (27.5%)	
Private gynecological monitoring			0.403
No	27 (26.2%)	23 (33.3%)	
Yes	76 (73.8%)	46 (66.7%)	
Folic acid supplementation			0.882
No	67 (67.0%)	42 (64.6%)	
Yes	33 (33.0%)	23 (35.4%)	
missing values	3	4	
Breastfeeding			0.791
No	18 (17.5%)	14 (20.3%)	
Yes	85 (82.5%)	55 (79.7%)	
Admission motive			0.866[1]
bleeding/3rd trimester hemorrhage	1 (1.0%)	1 (1.4%)	
CS	1 (1.0%)	0	
decrease fetal movements	1 (1.0%)	0	
oligohydramnios /anamnios	1 (1.0%)	0	
preeclampsia/suspicion of PE	1 (1.0%)	2 (2.9%)	
pre-labor	2 (2.0%)	1 (1.4%)	
premature or spontaneous rupture of membrane	14 (13.7%)	7 (10.1%)	
spontaneous/maturation/trigger	77 (75.5%)	58 (84.1%)	
suspicion of pathological cardiotocogram	3 (2.9%)	0	

Quality variables	Documented migrant non precarious (n=103, 59.9%)	Documented migrant precarious (n=69, 40.1%)	p-value (Mann-Whitney/chi^2)
termination of pregnancy/death in utero	1 (1.0%)	0	
missing values	1		
Timely US			0.756
No	8 (29.6%)	5 (21.7%)	
Yes	19 (70.4%)	18 (78.3%)	
missing values	76	46	
Appropriate time for first contact			-
missing values	103	69	

[1]Fisher's exact

Annex 4: Tables of results among precarious women with legal status, by migration status

Table 10: Socio-demographic variables for precarious women, Swiss vs. documented migrants

Socio-demographic variables	Swiss precarious SP (n=36, 34.3%)	Documented migrant precarious DMP (n=69, 65.7%)	p-value (Mann-Whitney/chi^2)
Age of patient			0.779
	31 (29 - 34.2)	32 (27 - 37)	
Parity			0.414
0	16 (44.4%)	26 (37.7%)	
1	8 (22.2%)	25 (36.2%)	
2+	12 (33.3%)	18 (26.1%)	
Gravidity			0.676[1]
1	11 (30.6%)	17 (24.6%)	
2+	25 (69.4%)	52 (75.4%)	
Tariff attribute			< 0.001[1]
no data	3 (8.3%)	4 (5.8%)	
Confederate domiciled in the canton	29 (80.6%)	0	
Foreigner domiciled in the canton	4 (11.1%)	63 (91.3%)	
Foreigner domiciled abroad	0	1 (1.4%)	
International officer/mission/consulate	0	1 (1.4%)	
French			0.956[1]
1st	23 (63.9%)	41 (59.4%)	
2nd	4 (11.1%)	9 (13.0%)	
no mention	9 (25.0%)	19 (27.5%)	
Marital status			0.337[1]
Single	12 (33.3%)	17 (24.6%)	

Socio-demographic variables	Swiss precarious SP (n=36, 34.3%)	Documented migrant precarious DMP (n=69, 65.7%)	p-value (Mann-Whitney/chi^2)
In a relationship (concubinage)	0	2 (2.9%)	
Married	21 (58.3%)	48 (69.6%)	
Separated/divorced	3 (8.3%)	2 (2.9%)	
Religion			0.382[1]
No religion	11 (30.6%)	13 (18.8%)	
Buddhism	1 (2.8%)	1 (1.4%)	
Christian	11 (30.6%)	19 (27.5%)	
Hinduism	0	2 (2.9%)	
Muslim	6 (16.7%)	15 (21.7%)	
Other	1 (2.8%)	1 (1.4%)	
Refusal to answer	2 (5.6%)	1 (1.4%)	
Not asked	4 (11.1%)	17 (24.6%)	
Profession			0.020[1]
No work	10 (27.8%)	32 (46.4%)	
Student	2 (5.6%)	0	
Domestic work	0	6 (8.7%)	
Low skill work	12 (33.3%)	17 (24.6%)	
High skill work	12 (33.3%)	14 (20.3%)	
Active smoker			0.999[1]
No	32 (88.9%)	59 (89.4%)	
Yes	4 (11.1%)	7 (10.6%)	
missing values		3	

[1]Fisher's exact

Table 11: Obstetrical variables for precarious women, Swiss vs. documented migrants

Obstetrical variables	Swiss precarious SP (n=36, 34.3%)	Documented migrant precarious DMP (n=69, 65.7%)	p-value (Mann-Whitney/chi2)
Term			0.249[1]
Preterm (<37 SA)	4 (11.1%)	4 (5.7%)	
Term (37-41 6/7 SA)	32 (88.9%)	65 (94.2%)	
Delivery type			0.489[1]
Spontaneous vaginal birth	21 (58.3%)	47 (68.1%)	
Instrumented vaginal birth	5 (13.9%)	9 (13.0%)	
Cesarean section	10 (27.8%)	13 (18.8%)	
Induced labor			0.779[1]
No	14 (38.9%)	28 (40.6%)	
Yes	16 (44.4%)	33 (47.8%)	
Cesarean section before labor	6 (16.7%)	8 (11.6%)	
Past CS			0.166
No	28 (77.8%)	62 (89.9%)	
Yes	8 (22.2%)	7 (10.1%)	
Single or multiple pregnancy			0.999[1]
No	35 (97.2%)	68 (98.6%)	
Yes	1 (2.8%)	1 (1.4%)	
Anesthesia			0.148[1]
No	5 (13.9%)	4 (5.8%)	
Gaz (EMONO)	0	3 (4.3%)	
Epidural	24 (66.7%)	51 (73.9%)	
General	0	4 (5.8%)	

Obstetrical variables	Swiss precarious SP (n=36, 34.3%)	Documented migrant precarious DMP (n=69, 65.7%)	p-value (Mann-Whitney/chi2)
Other/not described	7 (19.4%)	7 (10.1%)	
Postpartum hemorrhage			0.999
No	30 (83.3%)	58 (84.1%)	
Yes	6 (16.7%)	11 (15.9%)	
Perineal tear			0.630
None	22 (61.1%)	37 (53.6%)	
Type I	6 (16.7%)	17 (24.6%)	
Type II	8 (22.2%)	15 (21.7%)	
Episiotomy			0.489[1]
No	32 (88.9%)	64 (92.8%)	
Yes	4 (11.1%)	5 (7.2%)	
Threat of preterm delivery			0.999[1]
No	32 (97.0%)	62 (96.9%)	
Yes	1 (3.0%)	2 (3.1%)	
missing values	3	5	
Gestational diabetes or diabetes			0.102[1]
No	29 (80.6%)	64 (92.8%)	
Yes	7 (19.4%)	5 (7.2%)	
Gestational hypertension			0.178[1]
No	32 (88.9%)	67 (97.1%)	
Yes	4 (11.1%)	2 (2.9%)	
Eclampsia or preeclampsia			0.999[1]

**Obstetrical variables**	**Swiss precarious SP (n=36, 34.3%)**	**Documented migrant precarious DMP (n=69, 65.7%)**	**p-value (Mann-Whitney/chi2)**
No	34 (94.4%)	65 (94.2%)	
Yes	2 (5.6%)	4 (5.8%)	
Female genital mutilation			0.296[1]
No	33 (100%)	60 (93.8%)	
Yes	0	4 (6.2%)	
missing values	3	5	
Newborn’s birth weight			0.684[1]
Low (<2500 g)	3 (8.3%)	3 (4.3%)	
Normal (2500-3500 g)	31 (86.1%)	63 (91.3%)	
High (>3500 g)	2 (5.6%)	3 (4.3%)	
Newborn’s hospitalization in the neonatal unit - Main diagnosis			0.045[1]
none	31 (86.1%)	67 (97.1%)	
neonatal respiratory distress syndrome	1 (2.8%)	1 (1.4%)	
transient tachypnea in newborn	1 (2.8%)	0	
other neonatal respiratory distress	1 (2.8%)	0	
low birth weight	0	1 (1.4%)	
extreme prematurity	1 (2.8%)	0	
twin pregnancy	1 (2.8%)	0	

[1]Fisher’s exact

Table 12: Quality variables among precarious women, Swiss vs. documented migrants

Quality variables	Swiss precarious SP (n=36, 34.3%)	Documented migrant precarious DMP (n=69, 65.7%)	p-value (Mann-Whitney/chi2)
First contact with hospital: emergency room			0.694
No	24 (66.7%)	50 (72.5%)	
Yes	12 (33.3%)	19 (27.5%)	
Private gynecological monitoring			0.010
No	3 (8.3%)	23 (33.3%)	
Yes	33 (91.7%)	46 (66.7%)	
Folic acid supplementation			0.811
No	21 (60.0%)	42 (64.6%)	
Yes	14 (40.0%)	23 (35.4%)	
missing values	1	4	
Breastfeeding			0.362
No	4 (11.1%)	14 (20.3%)	
Yes	32 (88.9%)	55 (79.7%)	
Intention to breastfeed			0.418[1]
No	1 (2.8%)	6 (8.7%)	
Yes	35 (97.2%)	63 (91.3%)	
Admission motive			0.301[1]
bleeding/3rd trimester hemorrhage	0	1 (1.4%)	
preeclampsia/suspicion of PE	2 (5.6%)	2 (2.9%)	
pre-labour	0	1 (1.4%)	
premature or spontaneous rupture of membrane	6 (16.7%)	7 (10.1%)	
spontaneous/maturation/trigger	26 (72.2%)	58 (84.1%)	

Quality variables	Swiss precarious SP (n=36, 34.3%)	Documented migrant precarious DMP (n=69, 65.7%)	p-value (Mann-Whitney/chi2)
suspicion of pathological cardiotocogram	1 (2.8%)	0	
threat of preterm delivery	1 (2.8%)	0	
Timely US			0.999[1]
No	0	5 (21.7%)	
Yes	3 (100%)	18 (78.3%)	
missing values	33	46	
Appropriate time for first contact			-
missing values	36	69	

[1]Fisher's exact

Fig1

Click here to access/download;Figure;Figure1DistributionOfPatientGroups.tiff

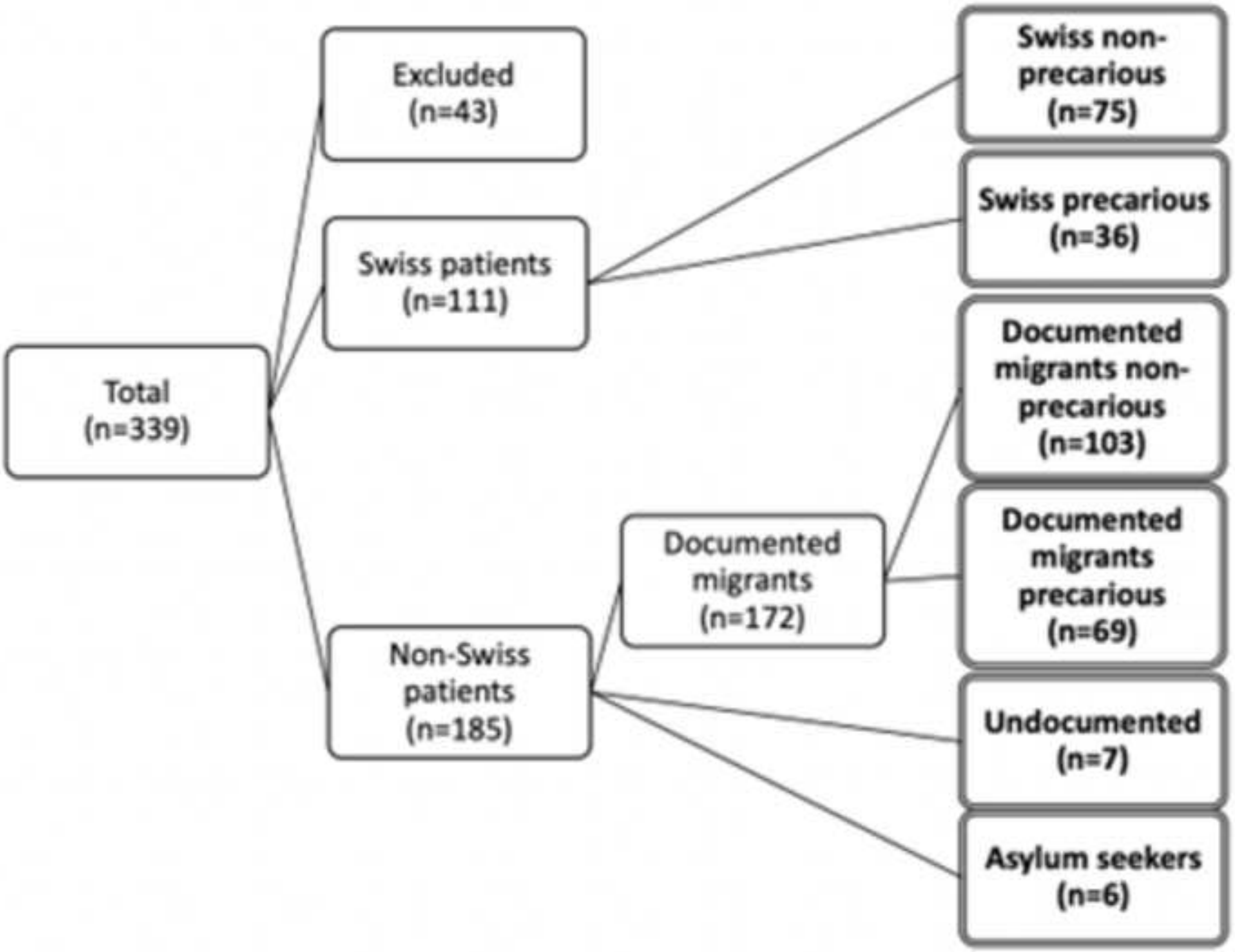

Fig2

Click here to access/download;Figure;Figure2NationalitiesOfWomenDelivering.tiff

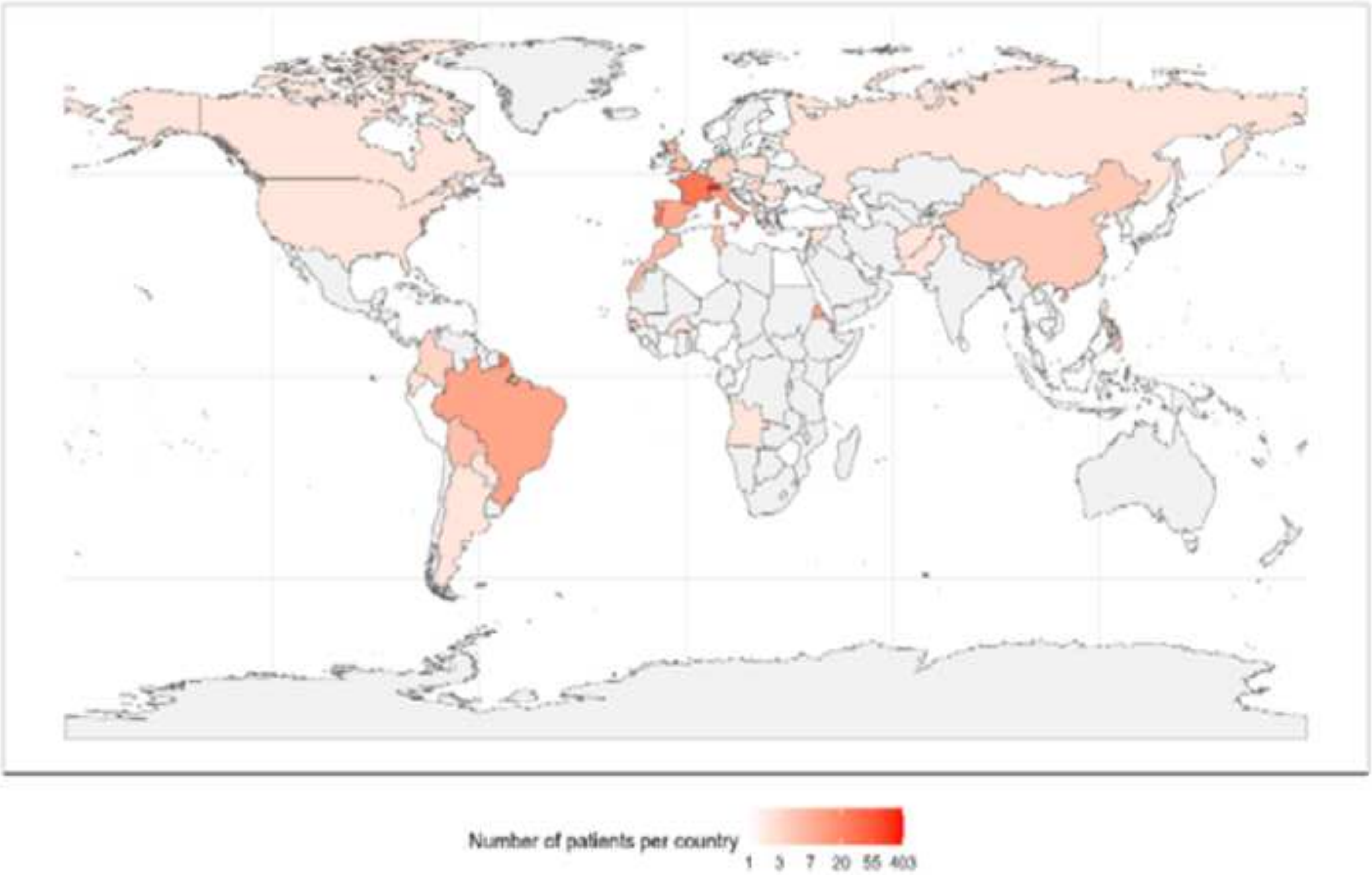

Fig3

Click here to access/download;Figure;Figure3TypesOfDelivery.tiff

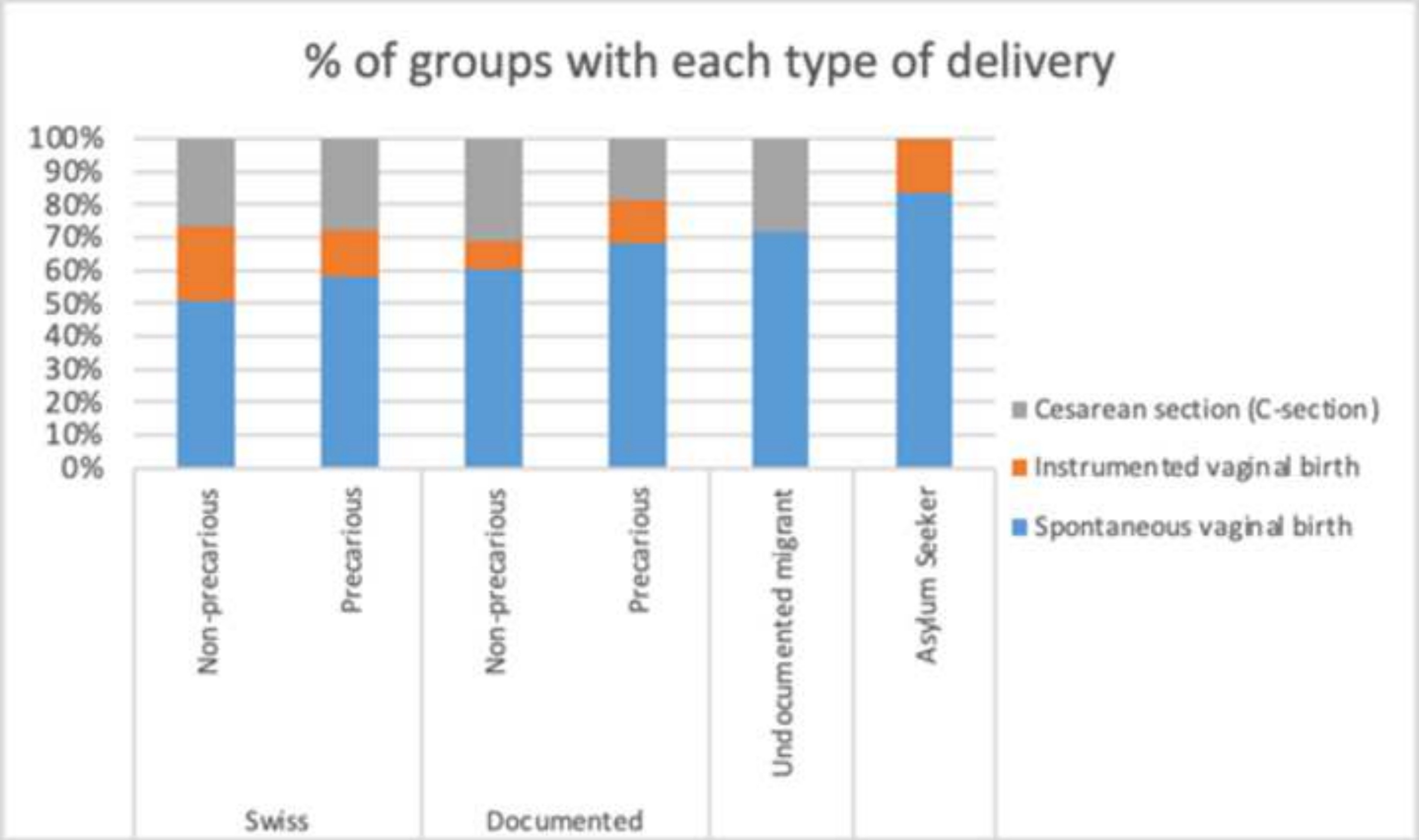

Fig4

Click here to access/download;Figure;Figure4LaborComplicationsVariables.tiff

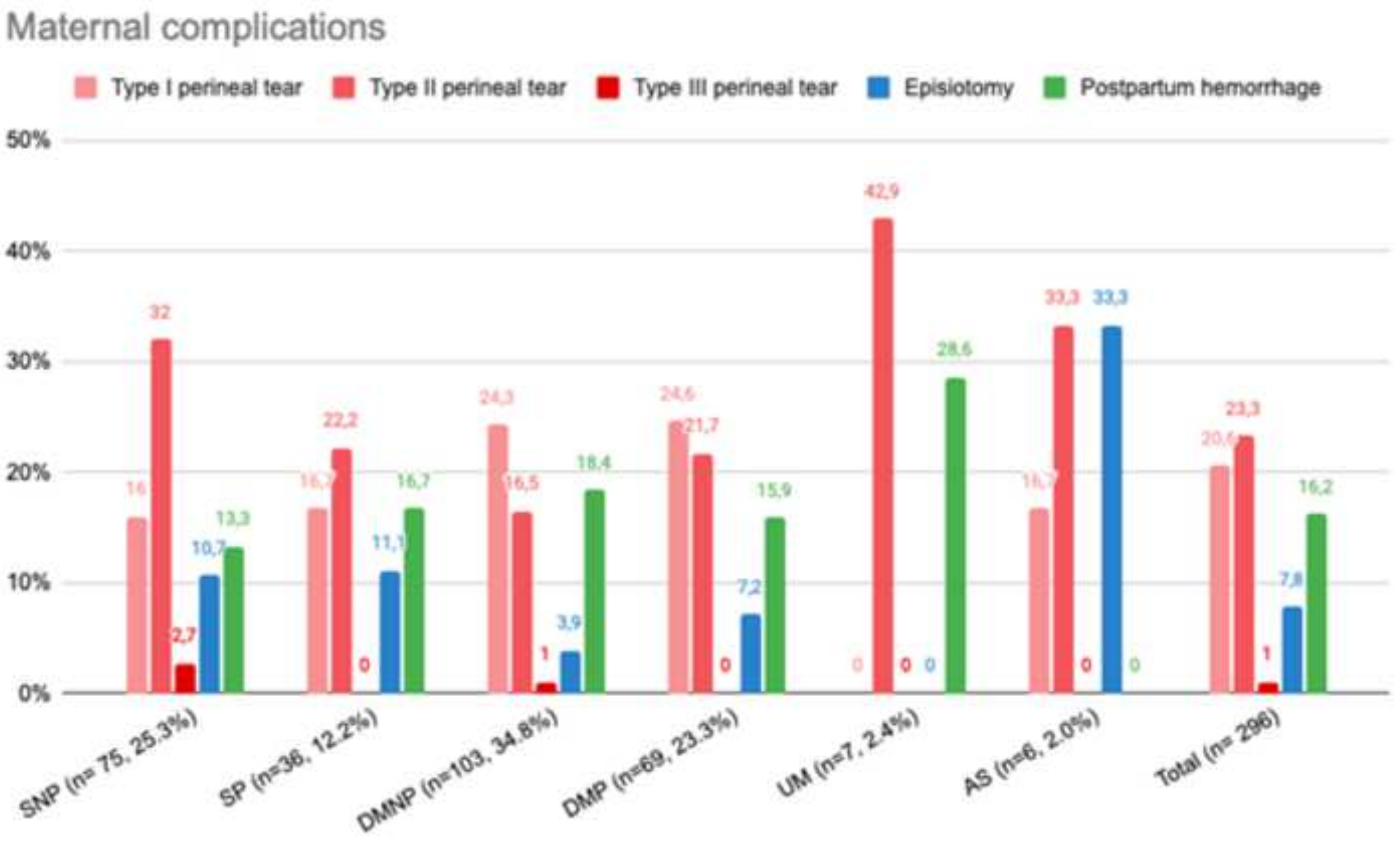

Click here to access/download;Figure;Figure5EntryPointofCareAtHUG.tiff

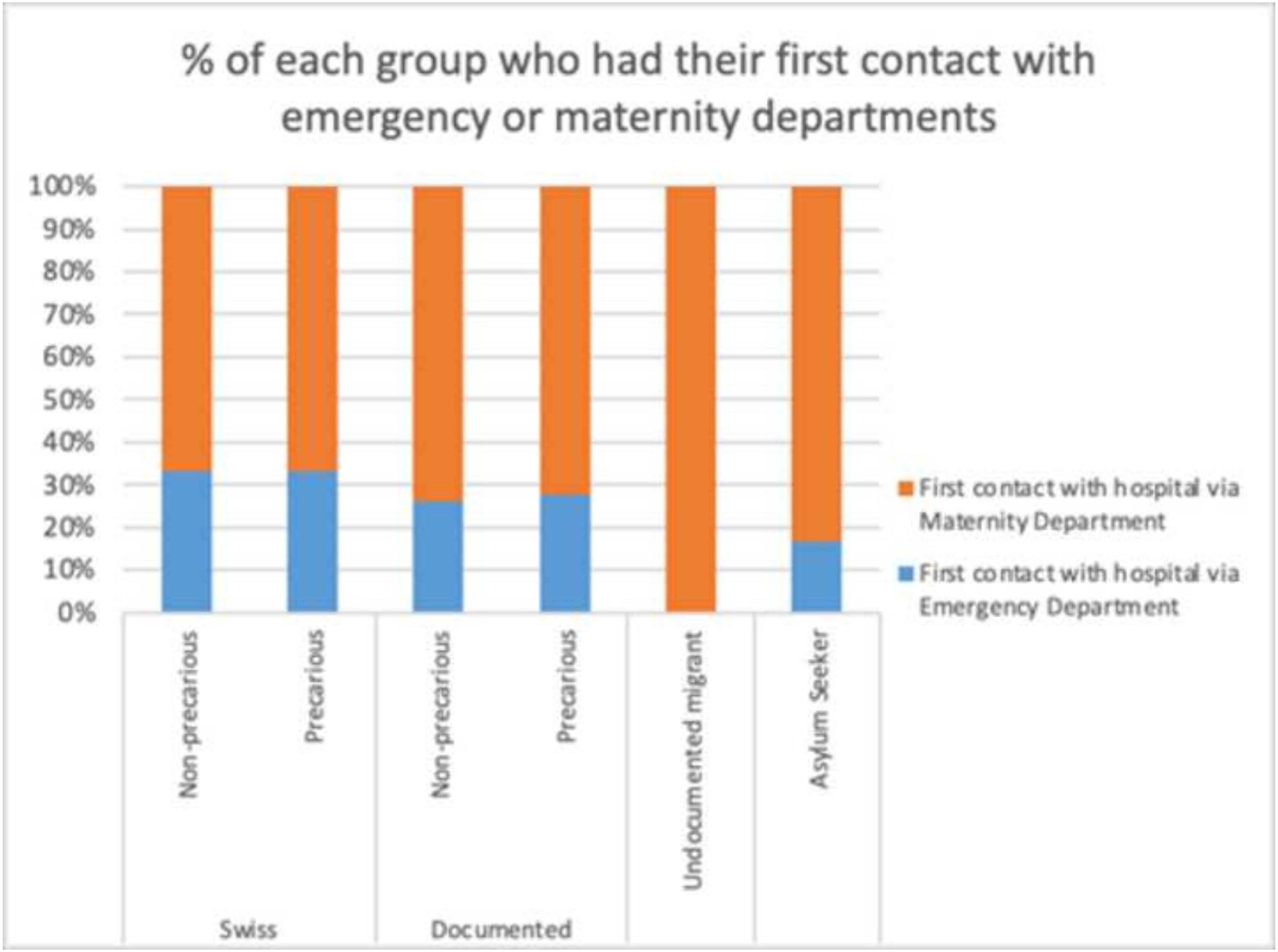

Supplement: Attachment — Submitted filename: PGPH-D-24-01793.pdf [file pgph.0004217.s014.pdf]
