# Supplementary material for: Influence of women’s legal status on pregnancy outcomes and quality of care: Findings from the Pregnancy of Migrants in Switzerland (PROMISES) program
Source: PLOS Glob Public Health. 2025 Apr 21;5(4):e0004217. doi: 10.1371/journal.pgph.0004217 (PMC12011233; doi:10.1371/journal.pgph.0004217)
Supplement: Attachment — Submitted filename: Review.docx [file pgph.0004217.s013.docx]

**Manuscript Number PGPH-D-24-01793
“Influence of women’s legal status on pregnancy outcomes and quality of care: Findings from the Pregnancy of Migrants in Switzerland (PROMISES) program”**

Thank you for the opportunity to review this manuscript. Overall, it is a well-constructed piece with potential for significant impact in its field. please consider my suggestions.

- **Abstract**

Results: Precarious patients represented 35.5% of the sample. Economic precarity was more highly associated with poor health outcomes than legal or migration status. **Please include the significance level in this section.**

- **Methodology**

Setting and study design
This exploratory cross-sectional retrospective study utilized existing data on all women who delivered at the HUG maternity department in May 2019. **Please include the sample volume in this section.**

- **References**

In the reference section, 8 to 9 of the references are quite old, dating back to 2004 or 2008 like the experience of an Italian hospital. Eur J Obstet Gynecol Reprod Biol. Oct 2004. **Please replace these with more recent references from 2010 onward.**
