# Supplementary material for: Influence of women’s legal status on pregnancy outcomes and quality of care: Findings from the Pregnancy of Migrants in Switzerland (PROMISES) program
Source: PLOS Glob Public Health. 2025 Apr 21;5(4):e0004217. doi: 10.1371/journal.pgph.0004217 (PMC12011233; doi:10.1371/journal.pgph.0004217)
Supplement: Attachment — Submitted filename: Response to reviewers.docx [file pgph.0004217.s016.docx]

**Response to reviewers for “Influence of women’s legal status on pregnancy outcomes and quality of care: Findings from the Pregnancy of Migrants in Switzerland (PROMISES) program”**

We would like to thank both reviewers for their thoughtful reviews and helpful suggestions.

| **Comment** | **Response** |
| --- | --- |
| *Reviewer #1* |  |
| Sample Size in Methods:  In the methodology section, the sample size is not clearly stated. I recommend explicitly mentioning the sample size used for the study to provide transparency and help readers assess the robustness of the findings. | In the Abstract, we have added the n of 339 to the first sentence in the Results section to reiterate it (it is previously mentioned in the first section of the Methods paragraph)  In the Methodology section of the main body of the paper, we have edited the first sentence to read:  This exploratory cross-sectional retrospective study utilized existing, routinely collected data on all 339 women who delivered a newborn (alive or dead, at any gestational age) women who delivered at the HUG maternity department in May 2019. |
| Results:  Precarious patients represented 35.5% of the sample. Economic precarity was more highly associated with poor health outcomes than legal or migration status. **Please include the significance level in this section.** | Which alpha to include?  We have added the following sentence in the “Results” section of the Abstract noting the study’s limitations:  Due to the small samples of undocumented migrants (UM) and asylum seekers (AS), the study had limited capacity to achieve statistical significance for findings. |
| Setting and study design:  This exploratory cross-sectional retrospective study utilized existing data on all women who delivered at the HUG maternity department in May 2019. **Please include the sample volume in this section.** | This sentence was amended to read:  This exploratory cross-sectional retrospective study utilized existing, routinely collected data on all 339 women who delivered a newborn (alive or dead, at any gestational age) at the HUG maternity department in May 2019. |
| References:  I noticed that several references are quite old, from 2004 or 2008. To ensure the relevance and currency of the research, please replace these older references with more recent ones from 2010 onwards. This will provide a stronger foundation for your study, reflecting the latest research and developments in the field.  In the reference section, 8 to 9 of the references are quite old, dating back to 2004 or 2008 like the experience of an Italian hospital. Eur J Obstet Gynecol Reprod Biol. Oct 2004. **Please replace these with more recent references from 2010 onward.** | We have replaced five dated references with more current articles and replaced an early paper from the Commission on Social Determinants of Health with its final report. The remaining older references are still relevant. |
| ***Reviewer #2*** |  |
| I have made the suggestion that vulnerability be included as a key word and in the title of the paper  (comment also inserted in PDF) | We have left the title as is, as legal migration status was the original focus of the study. However, we have added vulnerability as a **keyword** – thank you for that suggestion. |
| **Background:** Again, think vulnerability should be key word. (comment from PDF) | We agree. Vulnerability is first mentioned in the 4^th^ paragraph of this section and then used repeatedly afterwards. |
